# Same-gender citations do not indicate a substantial gender homophily bias

**DOI:** 10.1371/journal.pone.0274810

**Published:** 2022-09-20

**Authors:** Alexander Tekles, Katrin Auspurg, Lutz Bornmann

**Affiliations:** 1 Department of Sociology, University of Munich (LMU), Munich, Germany; 2 Science Policy and Strategy Department, Administrative Headquarters of the Max Planck Society, Munich, Germany; Shandong University of Science and Technology, CHINA

## Abstract

Can the male citation advantage (more citations for papers written by male than female scientists) be explained by gender homophily bias, i.e., the preference of scientists to cite other scientists of the same gender category? Previous studies report much evidence that this is the case. However, the observed gender homophily bias may be overestimated by overlooking structural aspects such as the gender composition of research topics in which scientists specialize. When controlling for research topics at a high level of granularity, there is only little evidence for a gender homophily bias in citation decisions. Our study points out the importance of controlling structural aspects such as gendered specialization in research topics when investigating gender bias in science.

## Introduction

Gender bias is an ongoing topic in science studies. There is evidence for various forms of gender differences, as a recent review in Science suggests: “Women have fewer publications … and collaborators … and less funding … and they are penalized in hiring decisions when compared with equally qualified men. The causes of these gaps are still unclear” [1]. At the same time, some studies report evidence against the existence of gender differences, e.g. with regard to funding [2–4] or hiring decisions [5, 6]. One question that has been frequently investigated hitherto is whether female scientists are cited less often by male scientists than by their female peers. The existence of such gender bias would imply disadvantages for female scientists. Citation scores are increasingly applied as a core metric to evaluate the performance of individual scientists as well as the quality of faculties, departments and institutional excellence at a global level [7]. Citations also matter for the distribution of resources, such as research grants or tenured positions [8]. To achieve gender equality in science, it is thus important to monitor possible gender gaps in citations and to understand their underlying reasons.

To date, literature shows mixed evidence of a possible gender citation gap. Some studies find no gender differences in citations or that female authors receive more citations than male authors [9–12], while other studies report that male authors receive more citations than female authors [13–15]. Be that as it may, gender homophily in citation decisions has been suggested as a reason for a possible gender citation gap where male authors receive more citations than female authors [16, 17]. We conceptualize gender homophily in citation decisions as the preference of scientists to cite other scientists *only* because they belong to the same gender category. We look at authors’ gender expression through names, but cannot distinguish this from authors’ gender identity which might differ. We also applied a binary concept of gender that only distinguishes between women and men. Thus, our analyses rely on a simplified concept of gender. Nevertheless, our analyses should provide a first insightful analysis of the extent to which preferences versus structural aspects lead to citation inequalities.

Our notion of homophily captures preferences that go beyond structural reasons for gendered citation patterns. Such structural aspects exist for example with the gendered specialization on research topics, which can result in male scientists citing more male-authored papers than female scientists (and vice versa). However, we conceptualize homophily as the “bias” that leads same gender peers to cite each other more often than what a baseline model of gender-blind selection of relevant literature would predict [18]. Evidence suggesting gender homophily in citation decisions has been reported for the fields economics [19, 20], anthropology [21], sociology [22], library and information science [23], communication science [24, 25], political science [26], and across different fields [17, 27–29]. See S2.1 in S1 File for details of these studies. Given the fact that more scientists are male than female, homophily in citation decisions alone could account for the observed citation advantage for male authors: as long as men are overrepresented in science, citing along gender lines would boost up citation scores of male scientists simply for the fact that they belong to the dominant gender group [16].

**Fig 1:**
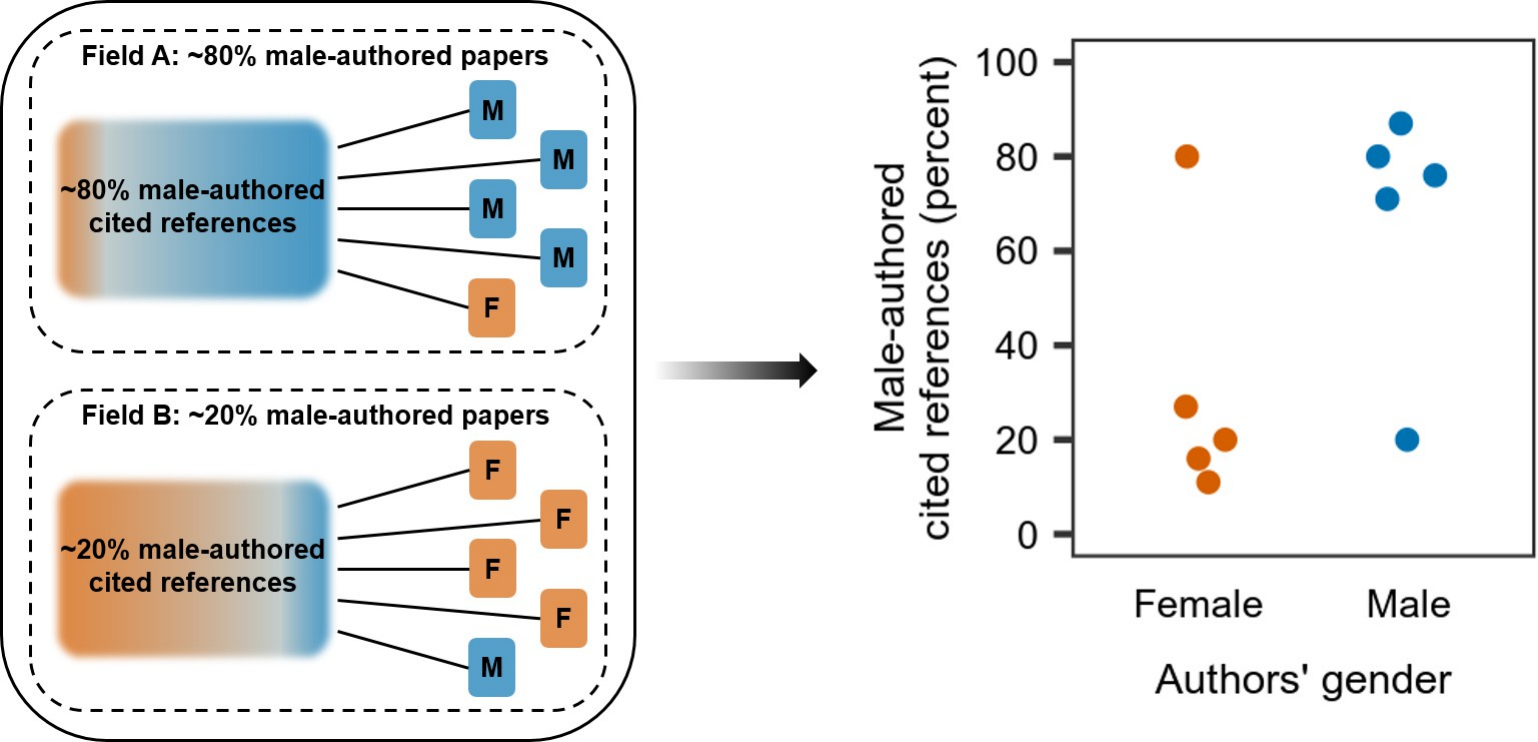
Schematic example illustrating the emergence of gendered citation patterns due to varying gender distributions across topics. On the left, female-authored and male-authored papers (denoted with “F” and “M” respectively) in two different fields and the gender distribution among their cited references are illustrated. Papers from one field are assumed to have one joint risk pool of papers to be cited. The plot on the right shows the resulting shares of male-authored cited references on the aggregated level. Even though this difference is solely based on the varying gender distribution across topics, it may be erroneously interpreted as a gender homophily bias in the authors’ citation decisions.

In our study, we tested the hypothesis that gendered citation patterns can emerge on the macro level due to structural aspects alone, with no gender homophily being at play. With gendered citation patterns, we mean the fact male scientists cite male-authored papers more often than female scientists (and vice versa) when looking at the pool of all scientists, regardless of their research area. Scientists’ gender is strongly related to the topic they are working on [14]. It follows that gendered citation patterns may result from varying gender distributions across different topics: whenever papers are pooled from discrete subfields that vary in their gender ratio, but which do not have one joint risk pool of papers to be cited for substantive reasons (e.g. due to their topic relevance), there will be a difference in the gender distribution among the cited authors between female and male scientists. Failure to control for the research topic as an important mediator between the gender of authors and gender distribution of cited references would then lead to an overestimation of homophily (Fig 1) [30].

To identify homophily bias, it is therefore important to control for structural aspects that make some papers to more adequate sources to be cited than other papers. Besides research quality, the most important structural aspect to define such risk pools of papers is certainly topic similarity (overlap in research questions, theories and/or methods). Previous homophily studies have already tried to control for this topic similarity by considering the journals authors have published in [17, 19–28]. However, it is questionable whether this sufficiently controls for topic similarity, because journals often accept work from different subjects that show little or even no overlap in research topics or methods. Ghiasi, Mongeon, Sugimoto and Larivière [29] used more information on the papers’ content to identify topic similarity by matching papers that appeared in the same issue of the same journal based on the papers’ abstract and title. But matching only within journal issues may have restricted the ability to identify papers that are similar due to the small number of papers: it is highly probable that more similar papers are available beyond the journal issue.

Our goal was therefore to test more rigorously whether gendered citation patterns are caused by a gender homophily bias. To precisely control for the risk pool of papers to be cited, we measured the topic similarity between papers based on (the combination of) keywords that were assigned manually by experts. We drew on data provided by Faculty Opinions (https://facultyopinions.com/; previously F1000Prime) that contains this information for papers published in 2002–2020. The keywords assigned to these papers were curated by an editorial team at Faculty Opinions in cooperation with leading scientists and clinicians in the corresponding biological and medical research fields. For each of the papers, the data also include information from reviews of experts in these fields [31]. We were able to classify the gender of all authors (using a binary coding distinguishing typical male and female first names) of ~38,500 papers in that database along with ~335,000 papers that subsequently cited these focal papers. We validated the results with Web of Science (WoS) data (including almost 400,000 papers across all scientific disciplines) and alternative approaches to measure topic similarity. By controlling the topic similarity between papers at different levels of granularity, we were able to study how empirical results on gender homophily are influenced by the approach to control the papers’ similarity.

Our main finding is that thoroughly controlling for research topic is important for validly assessing the degree of gender homophily. The level of observed gender homophily substantially decreases, the more fine-grained measurements of topic similarity are used. At a high level of granularity, only very little evidence remains for a possible homophily bias. We conclude that although gender homophily may affect citation decisions to some degree, the impact of this bias has likely been overestimated in the literature due to insufficient controls for topic similarity to define potential pools of papers to be cited in different research areas.

## Results

### Results on biomedicine with Faculty Opinions data

**Fig 2:**
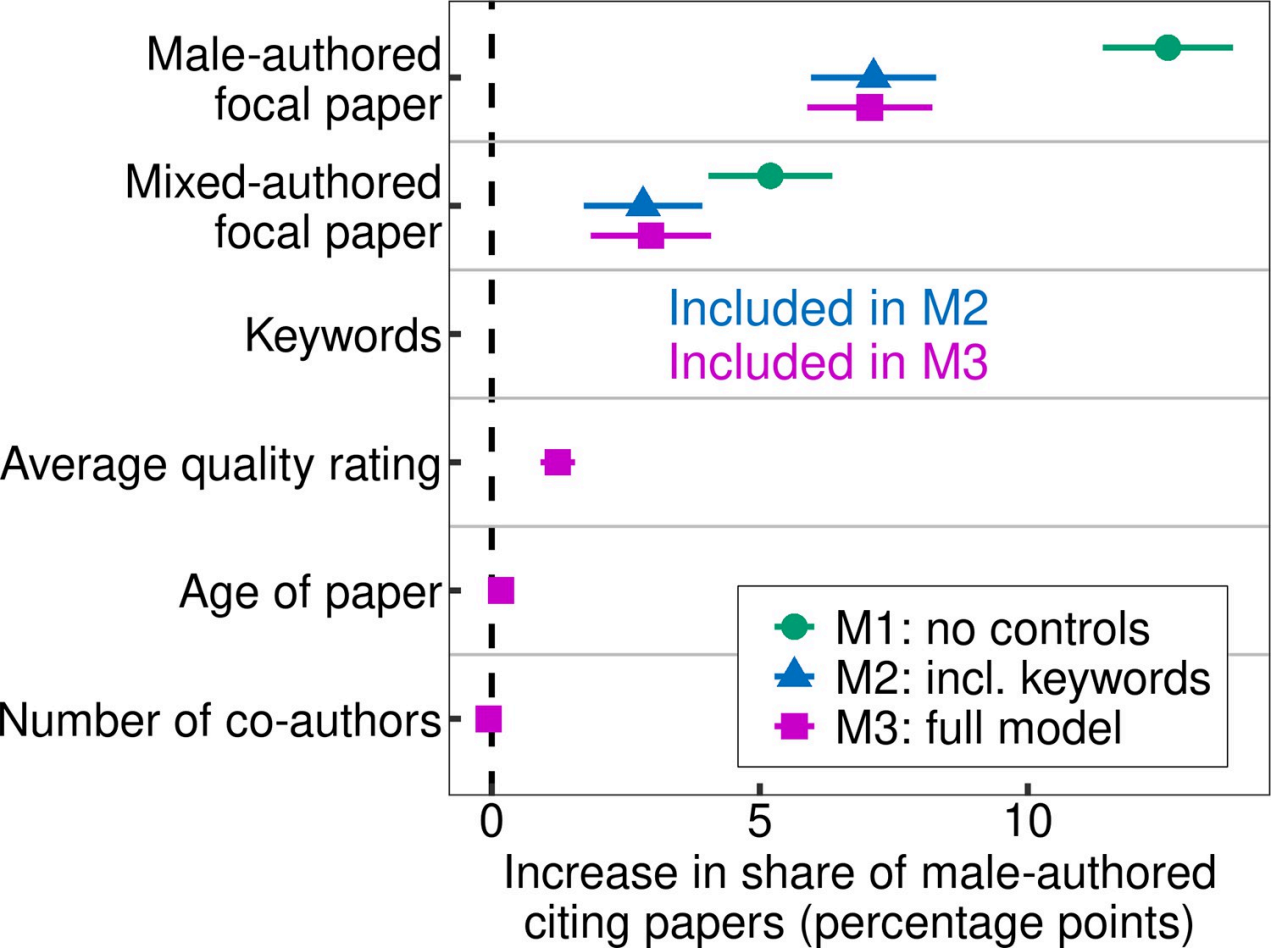
Marginal effects (with 95% confidence intervals) of three regression models on the level of focal papers. For each model, the dependent variable is the share of male-authored papers among the citing papers. In addition to the gender of the focal papers’ authors, we successively included as possible structural factors the focal papers’ keywords for research topics (in the form of binary variables to control for all 334 keywords in the Faculty Opinions database), the quality rating (average quality rating in case of quality ratings by multiple experts for one paper), the age of the papers (publication year), and the number of authors. All models are based on 38,439 observations (focal papers). For more information and detailed analyses, see S1.3 in S1 File.

Fig 2 shows the results of a linear OLS regression using the papers included in the Faculty Opinions database and their metadata as observations. Table 1 shows the coefficient estimates for the regression analyses. The dependent variable is the share of male-authored papers among the citing papers. Note that we used the focal papers’ citing papers instead of their cited references, as other studies have done. This allowed for a better control of the publication year of the papers on the cited side (the focal papers in our case). This is necessary to control for the gender composition of authors: the gender distribution in science has changed over time, which, if not taken into account, could also artificially lead to evidence of homophily bias when male authors are more likely to work in fields whose literature appeared earlier (see S2.2.3 in S1 File). The main independent variable is the gender of the focal papers’ authors, whose direct effect can be interpreted (once all indirect effects arising from structural aspects are controlled) as the degree of gender homophily in citation decisions: if there was a gender homophily bias in citations, the share of male-authored papers among the citing papers would be higher for male-authored focal papers than for female-authored focal papers. To facilitate a clear interpretation of the results, we focused on the comparison between female-only and male-only author teams in our analyses and included other papers as “mixed-authored.” We excluded all self-citations (i.e., citations where citing and focal paper share at least one author name), since they artificially increase the correlation between the gender of the focal and citing papers’ authors.

**Table 1:** Results for the regression models on the level of focal papers.

	Dependent variable: share of male-authored citing papers		
	M1	M2	M3
Gender of focal papers’ authors (reference category: female)			
Male	12.613***	7.123***	7.053***
	(0.620)	(0.596)	(0.596)
Mixed	5.198***	2.823***	2.969***
	(0.590)	(0.565)	(0.573)
Faculty Opinions keywords		Included	Included
Quality rating (average)			1.232***
			(0.164)
Age of paper			0.175***
			(0.034)
Number of authors			-0.059*
			(0.026)
Intercept	23.115***	26.281***	23.581***
	(0.579)	(0.588)	(0.695)
*N*	38,439	38,439	38,439
*R* ^2^	0.029	0.136	0.138

Note. Regression estimates underlying Fig 2. Robust standard errors in parentheses.

* *p* < 0.05

** *p* < 0.01

*** *p* < 0.001 (two-tailed tests).

Model M1 (green) shows that for male-authored focal papers, the share of male-authored citing papers is about 12.6 percentage points higher than for female-authored focal papers. However, no further variables are included in this model. Model M2 (blue) shows that this effect reduces to about 7.1 percentage points when controlling for keywords (in the form of binary variables for all keywords in the Faculty Opinions database). This means that a gender-specific selection of scientists into different topics is–at least partly–responsible for the observed gendered citation patterns. Controlling for further factors (average quality rating, age of paper, and number of authors) in Model M3 scarcely changes the effect of the gender of the focal papers’ authors. We controlled for publication year and number of authors, since empirical analyses suggest that the share of female authors increased over time [32], and that female authors have fewer co-authors than men [9, 33].

In line with some previous studies [17, 27, 29], these results suggest that controlling for topics is necessary in order to not overestimate the degree of gender homophily preferences in citations. The results also reveal that a certain degree of homophily remains even after controlling for topic.

**Fig 3:**
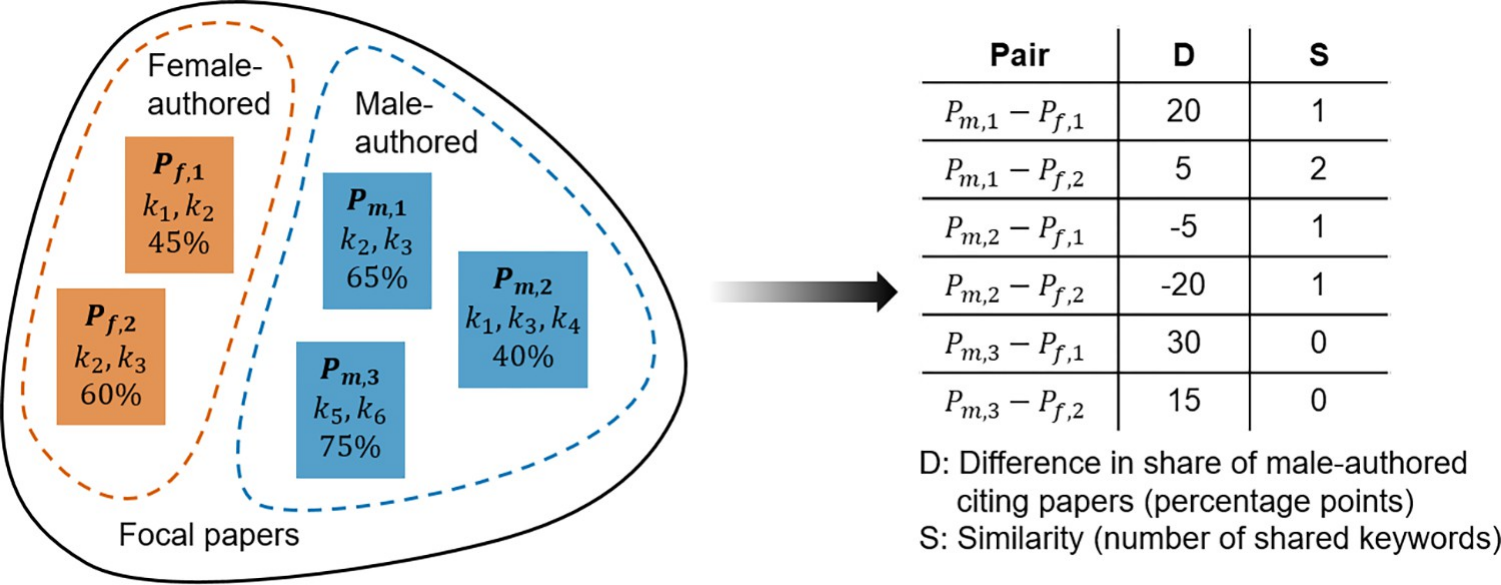
Generating pairs of focal papers. On the left, five focal papers are illustrated, together with their keywords (k_i_) and the share of male-authored citing papers (in %). The table on the right shows all pairs of focal papers such that one paper of a pair is female-authored and the other paper is male-authored. Column D shows the difference in the share of male-authored citing papers for a pair, which is used as an indicator for the degree of gender homophily. Column S shows the number of shared keywords, which is used as an indicator for the similarity between two papers.

However, the inclusion of keywords in the form of binary variables in a regression model only allows controlling for each keyword independently of other keywords. Since research is usually reflected by more than one keyword (on average, 11 keywords are assigned to a paper in the Faculty Opinions dataset), topics may be better represented by certain (dependent) combinations of keywords. To consider this, we generated pairs of focal papers such that one paper is authored only by male scientists and the other paper is authored only by female scientists (see Fig 3). For each pair, we used the number of shared keywords as a measure for the similarity between the two papers. The difference in the share of male-authored papers among the citing papers that remains after controlling for topic similarity (measured on different levels of granularity) serves as an indicator for gender homophily. Using these differences, we plotted histograms for all pairs of focal papers with at least *X* shared keywords. With increasing *X*, the pairs are increasingly similar in terms of keywords (describing both focal papers’ research topic).

**Fig 4:**
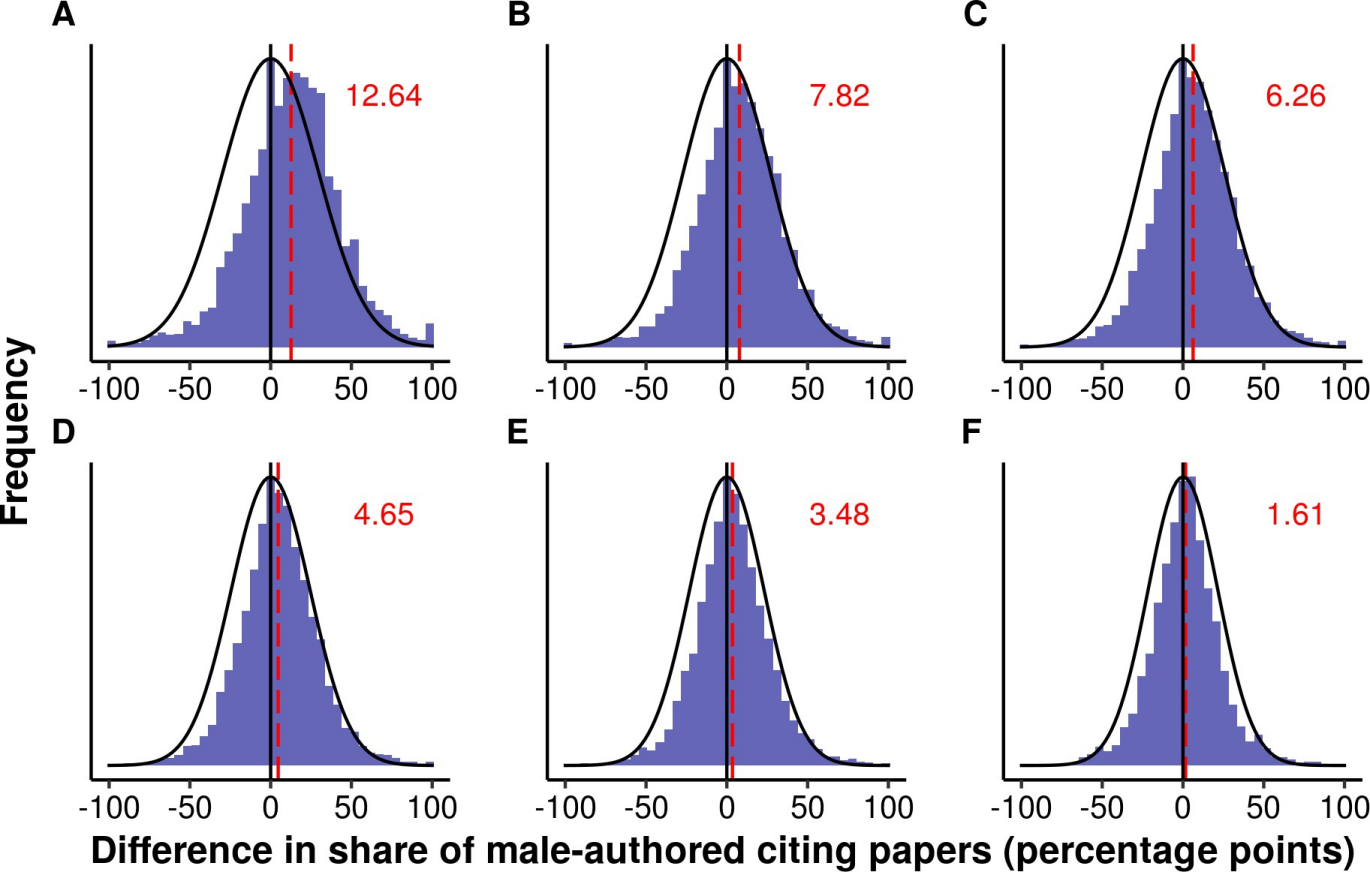
Histograms for the differences in the share of male-authored citing papers for pairs of focal papers (Faculty Opinions). In each histogram, the pairs of focal papers are restricted to those cases in which one focal paper is authored only by male scientists and the other focal paper is authored only by female scientists. Positive differences result when the male-authored paper of a pair has a higher share of male-authored citations than the female-authored paper of this pair. The histograms differ in terms of the minimum number of shared keywords that the pairs of focal papers have, and–as a consequence–in the number of pairs of focal papers included: all 11,702,080 pairs in (A), 765,642 pairs with at least one shared keyword in (B), 223,837 pairs with at least two shared keywords in (C), 58,465 pairs with at least three shared keywords in (D), 14,167 pairs with at least four shared keywords in (E), and 3,010 pairs with at least five shared keywords in (F). The vertical lines are placed at 0 (black) and at the observed average difference (red, dashed). The black curve shows the shape of a normal distribution.

Fig 4 shows that the average difference in the share of male-authored citing papers between male-authored and female-authored focal papers is positive, meaning that male-authored focal papers are more likely to receive their citations by male authors than female-authored focal papers. But for increasing *X* (i.e., topic similarity), the difference approaches the shape of a normal distribution. The shape of a normal distribution could be expected if there is no gender homophily in citations: with no homophily bias, on average, the difference in the share of male-authored citing papers would be zero, and the differences would be distributed symmetrically around this average (with cases becoming the less frequent, the larger the distance to this zero-difference reference line). These results suggest that after controlling for the topic on a sufficiently high level of granularity (i.e., beyond the inclusion of keywords in the form of binary variables), gender homophily can be scarcely observed.

### Extension to other research fields and data

We deem the keywords in the Faculty Opinions database a reliable approach for measuring the topic similarity between papers, since these keywords are based on expert knowledge and provide a more consistent measurement compared to keywords idiosyncratically chosen by authors [34]. Although this information is a particular advantage of our dataset, the dataset is restricted to biological and medical areas and research of outstanding quality [35].

**Fig 5:**
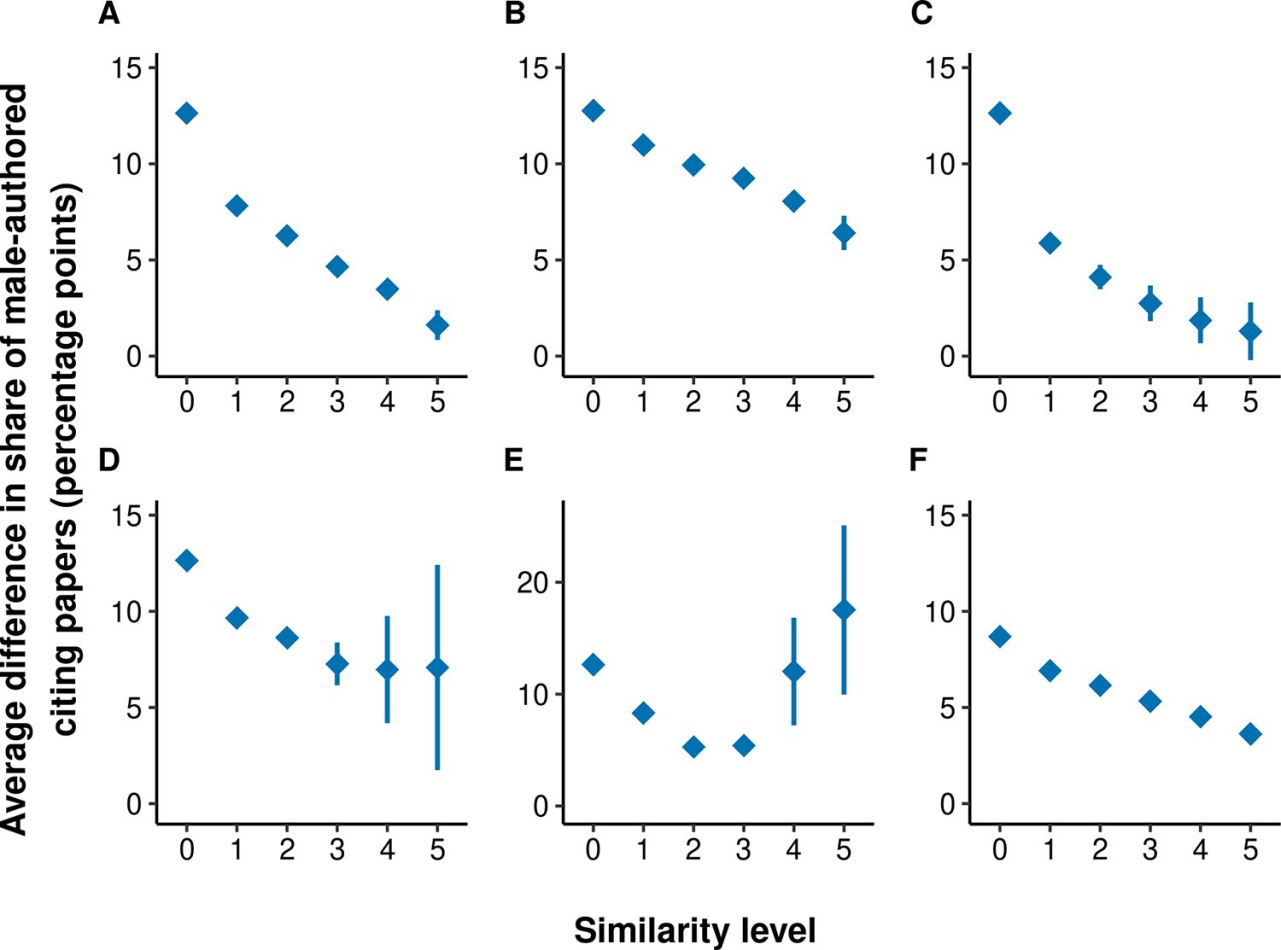
Results for alternative approaches to measure similarity. Average differences in the share of male-authored citing papers across different similarity levels. Bars show 95% confidence intervals. (A)-(E) are based on Faculty Opinions data, (F) on WoS data. The similarity between focal papers is measured using the number of shared keywords provided by the Faculty Opinions database in (A), abstracts and titles in (B) and (F), cited references in (C), keywords provided by the WoS in (D), and WoS subject categories in (E). For (C)-(E), the similarity levels represent the number of shared keywords, cited references or subject categories. For (B) and (F), the similarity levels are specified such that the share of pairs of focal papers corresponds to these shares in (A), see S2.2.5 and S7-S11 Figs in S1 File for detailed results.

We therefore tested whether our results also hold for alternative similarity measurements (Fig 5A–5E) and a set of focal papers covering a broader range of fields than the Faculty Opinions dataset (Fig 5F). For each similarity measurement, we defined six similarity levels, according to the number of shared Faculty Opinions keywords used in the results shown in Fig 4. All of these analyses confirm the main result: the degree of observed gender homophily decreases as the similarity between papers is controlled for more thoroughly. However, the remaining gender effects indicating homophily are generally slightly larger than when using the keywords provided in the Faculty Opinions database (see also S2.2.5 in S1 File). The most plausible explanation for this result is that the alternative approaches for measuring the similarity between papers provide less precise measures of topic similarity than the more standardized assignment of keywords by experts. This insufficient control for citation pools may induce spurious evidence of gender homophily.

For identifying comparable pairs of papers, the approach based on titles and abstract is the most efficient alternative to using Faculty Opinions keywords (this also manifests in the relatively large confidence intervals for the other approaches). Therefore, we used this approach to expand the analyses to all papers from the WoS published in 2012 (the mean publication year for the papers in the Faculty Opinions dataset). For these data, the observed degree of gender homophily is generally smaller than for the Faculty Opinions data. Since these results are based on the less precise approach to measure topic similarity based on titles and abstracts, we can be even more sure that there is no marked gender homophily that goes beyond gender compositions of research fields.

We were able to replicate our results in various further robustness checks (e.g., including more controls, using alternative statistical models or using female- instead of male-authored citing papers to measure gender homophily; see S2.2 in S1 File). A noteworthy side result of these checks is that both, not excluding self-citations and using cited references instead of citing papers for measuring the degree of homophily (which means a limited control of papers’ age), inflate the observed gender homophily. This suggests that the gender homophily reported in the literature is also inflated by those design aspects: only some existing studies excluded self-citations and none used citing papers to measure homophily.

## Discussion

Just as in previous studies, we were unable to conduct a randomized experiment and instead had to rely on large-scale bibliometric data. The main takeaway from our study is the necessity of using adequate measures for controlling mediating factors when studying gender bias: only when all relevant mediators are controlled with exact measurements can the *genuine* gender bias that defines homophily be identified. Our study reveals the importance of one mediating factor in particular: the research topic. Without controlling the topic at a fine-grained level, this study would have erroneously concluded (as did others) that there is a strong homophily bias. The very small evidence for homophily bias that remains in our study after controlling for topic similarity suggests that other mediators are not very meaningful. Since previous studies have shown that more productive, senior authors collect more citations [36], seniority might be a possible meaningful mediator for homophily. Based on our results, however, seniority can be excluded as meaningful mediator.

Similar to previous studies on gender homophily in citations, our approach to identify the authors’ gender based on their first names implies an imprecise concept of gender. It is unclear to what extent this approach measures only a person’s gender expression and not their possibly different gender identity. Thus, our results do not allow to differentiate between these notions of gender. Our approach to infer the scientists’ gender is also limited to a binary concept of gender, which means that we cannot draw any conclusion about scientists with non-binary gender. Future research could address these issues by applying a more differentiated concept of gender.

Gender (homophily) bias is also suspected in many other realms of science, including reviews of publications, grant assignments or decisions to select co-authors or peers for acknowledgments [30, 37, 38]. Reliable measures of research topics (and other possible sources of gender heterogeneity) are needed not only to rule out mediators in these realms as well, but also to achieve sufficient statistical power to detect genuine gender bias that may still exist in many realms (decisions) in science [39]. Developing measurements of research field-specific clustering is therefore an important topic (in bibliometrics) for investigating gender bias. So far, there is no robust and generally accepted standard solution [40]. Our results suggest combinations of keywords assigned by experts to be a promising approach, at least to measuring risk pools that underlie citation decisions.

Our study also points out, in accordance with many other empirical studies [e.g., 41–44], that there are structural mechanisms other than gender homophily leading to gender differences in citations. Also many other studies found no evidence for a genuine gender bias in science once they controlled for structural factors, such as different career lengths or qualifications [3–5, 11, 42, 45]. In a recent blog post, Traag and Waltman [46] emphasize the importance in gender bias studies of understanding the underlying causal mechanisms. Only by uncovering the micro-mechanisms actually producing the gender differences observed on the macro level can effective measures be proposed to mitigate them. Our results indicate that the sorting of female and male scientists into different fields and topics [which has been shown, for example, by 32, 47, 48] is one of the most important mechanisms producing gendered citation patterns on the macro level. Therefore, one should in particular research the mechanisms underlying gendered specializations in research topics (so-called “horizontal segregation”), whether due to self-selection or sorting by gatekeepers.

## Materials and methods

The Faculty Opinions data that we used in this study includes expert ratings of the papers’ scientific quality, which are given in the form of "good," "very good," and "excellent." Thus, only papers at a high quality level were selected for inclusion in the database. Information about the topic of the papers is given in the form of keywords assigned by experts (an editorial team at Faculty Opinions in cooperation with leading scientists and clinicians in the corresponding biological and medical research fields). There are 334 different keywords occurring in the database, and an expert may have assigned multiple of these keywords to a paper. Since keywords and quality ratings have been assigned by experts in the field (and in many cases by more than one expert per paper), we can assume a high accuracy of the data.

We matched the papers in the Faculty Opinions database (focal papers) with metadata on authors and topics from the WoS. From these data, we used the author names (to infer the authors’ gender) and the publication year for both the focal papers and all of their citing papers. For this purpose, we used an open source application for assigning a gender category (female or male) to first names [49; see also S1.2 in S1 File]. At the paper level (for both focal and their citing papers), we operationalized the authors’ gender in the form of three categories: all co-authors are female, all co-authors are male, or the team consists of both female and male co-authors. In the regression analyses, we included the gender of the focal papers’ authors in the form of two dummy variables for the categories indicating male-authored focal papers and mixed-authored focal papers, with female-authored focal papers as reference category.

## Supporting information

S1 File (PDF)
