# Supplementary material for: Same-gender citations do not indicate a substantial gender homophily bias
Source: PLoS One. 2022 Sep 20;17(9):e0274810. doi: 10.1371/journal.pone.0274810 (PMC9488760; doi:10.1371/journal.pone.0274810)
Supplement: S1 File — (PDF) [file pone.0274810.s001.pdf]

Supplementary Information for
Same-gender citations do not indicate a substantial gender homophily bias

Alexander Tekles, Katrin Auspurg, Lutz Bornmann

Corresponding author: Alexander Tekles
Email: alexander.tekles.extern@gv.mpg.de

S1. Materials and methods

S1.1. Dataset

For the analyses presented in Fig. 2, Fig. 4, and Figs 5A-5E, we used papers that are included in the Faculty Opinions database and for which we could merge the necessary metadata from the Web of Science (WoS) database. The Faculty Opinions database initially contains information for 162,071 papers. Due to missing metadata and restrictions in our analyses, we could not include all these papers in our analyses. Fig. S1 illustrates how many papers had to be excluded at each step in the data preprocessing phase. Most papers had to be excluded due to insufficient information on the authors' gender (for at least one author of a paper, the gender could not be inferred). We decided to use only reliable gender information, although this reduced the number of papers that could be included in the analyses. For a large share of authors, the gender could not be inferred because no first name or only the initials of the authors' first name(s) are given. Of all authors with missing gender information, 1.9% have no author name provided in the WoS, 28.6% have only one letter given as first name string, and another 19.3% have only two letters given as first name string. This suggests that for at least one-third of the authors the missing gender information is due to a missing full first name: for almost all cases with only one letter (and for most cases with two letters) given, it can be assumed that these are the first names' initials.

On the paper level, we labelled the gender as missing if at least one author has missing gender information. Among the papers with missing gender information, 36.7% have at least one author with missing name and 73.5% at least one author with unisex name (see S1.2 for more information on how the authors' gender was classified).

Further analyses show that information on gender is missing for papers with older publication dates in particular. Papers with sufficient gender information have on average been

published in 2012, while papers with insufficient gender information have on average been published in 2011. This can be expected due to the increasing availability of full first names in the WoS over time. Since even one author with missing gender information results in no gender being assigned to a paper, papers with multiple authors are more likely to have missing gender information. This is also reflected in our data: papers that could be assigned a gender category were written on average by fewer authors (arithmetic mean: 6; median: 5) than papers that could not be assigned a gender category (arithmetic mean: 10; median: 7). Papers without gender information also have more citations on average (arithmetic mean: 126; median: 57) compared to papers with gender information (arithmetic mean: 92; median: 43). However, this difference reduces and reverses (papers without gender information receive on average six citations less than papers with gender information) once the publication year and number of authors are controlled for. This means that missing gender information is only slightly related to citation counts independently of publication year and number of authors.

In our regression analyses (models M1-M3), we compared male- and mixed-authored with female-authored focal papers. Including also mixed-authored papers is one advantage of this approach (besides the possibility to use control variables). For mixed-authored papers, the share of male-authored citing papers is larger than for female-authored papers, but smaller than for male-authored papers; thus, dropping mixed-authored papers (as has been done in other analyses) yields an upper bound of gender effects on citations. We were able to include 38,439 papers in our analyses (few cases were lost due to missing information on the Faculty Opinions keywords that we used as controls, see Fig. S1). We controlled for the Faculty Opinions keywords by single dummy variables. These dummies measure topic similarity only on a low level of granularity: we controlled for single keywords, but not for idiosyncratic combinations of keywords that would

define research topics more precisely (but see S2.2.1 for some robustness analyses with regressions based on pairs of papers that allow for more fine-grained controls of papers' similarity).

Therefore, we complemented regressions including single dummy variables with a novel approach based on pairs of papers. The alternative approach allowed us to control the papers' topic not only at a more fine grained level, but also at different levels of granularity. For these analyses presented in Figs. 4-5, we paired papers of female- and male-authored papers. We could not include mixed-authored focal papers in the analyses. While pairing the focal papers increases the number of observations (different pairs of papers are observed instead of single papers), it reduces the number of focal papers that we could consider due to the necessary omission of mixed-authored papers. From the 10,541 papers that we included in the analyses based on pairs of papers, 1,261 have only female authors and 9,280 only male authors. Building all possible pairs of papers such that one paper is authored only by female scientists and the other paper is authored only by male scientists results in 11,702,080 pairs that we could use for plotting the histograms in Fig. 4.

In the analysis presented in Fig. 5F based on WoS data, we considered all papers that are included in the WoS, have been published in 2012, and are of document type 'article' or 'review' (to include only substantial papers). This comprises 1,396,207 papers in total. Due to missing metadata, the set reduces to 1,235,021 papers. For 1,212,097 of the papers, the title and abstract are available in the WoS. The gender of all authors could be classified for 399,319 papers (117,143 with only female authors and 282,176 with only male authors). When matching the pairs, we only considered pairs for which both papers are in the same WoS subject category. This drastically reduces the computational complexity and makes the results more comparable to those based on the Faculty Opinions data: the papers in the Faculty Opinions database are field-specifically

restricted (to biomedicine). We were able to build 508,941,740 pairs of papers with one being female-authored and the other being male-authored.

S1.2. Determining gender of names

In order to infer the authors' gender (of both focal papers and their citing papers), we used an open source application that makes it possible to assign the gender to first names [1]. This application is based on a list of 44,568 first names that have been mapped to a gender, depending on the country of origin. In order to consider the country of origin when inferring the authors' gender, we used the authors' affiliation as a proxy. In the case of unisex names, the application returns no gender to a given first name (in combination with a country of origin): the name may refer to female, male, or non-binary persons. If a first name is usually associated with a particular gender in a country, but is also used for the other gender in another country, the application classifies this name as probably female/male. These cases are included in our analyses, i.e. a probably female (male) author is assumed to actually be female (male). Fig. S6 shows the results for the pair-based analyses using the Faculty Opinions data when probably female/male authors are excluded and only the more reliable gender assignments are used. Since these results do not differ substantially from the results obtained when including the less reliable gender assignments, we conclude that both approaches can be used interchangeably for our analyses.

In order to operationalize the gender of author teams, we distinguished three cases: all authors are female, all authors are male, and the authors are of mixed gender. If we could not infer the gender of at least one author (because it is a unisex name or the name is not in the application's database), the paper is not included in our analyses. If multiple affiliations in different countries are linked to an author, we determined the gender of the author separately for each affiliation. If

the gender classifications match, the paper remains in the analyses; if there are inconsistencies across the different classifications, the paper is not included in the analyses.

S1.3. Regression analyses

We tested the regression models presented in Fig. 2 and Table 1 for heteroscedasticity, multicollinearity, and outliers. To test for heteroscedasticity, we performed Breusch-Pagan tests [2]. The tests are statistically significant on the 0.001 level for all three models, indicating that the variance of the error terms depends on the values of the independent variables. Therefore, we used robust standard errors for calculating p values in Table 1 and confidence intervals in Fig. 2. To test for multicollinearity, we calculated the variance inflation factor (VIF) for the independent variables in all models. For the dummy variables indicating the gender of the focal papers' authors, we calculated the generalized VIF (GVIF) [3]. GVIF accounts for the fact that the two dummy variables represent the same characteristic. Since the VIF/GVIF is smaller than five in every case, we assume a negligible level of multicollinearity in the models [4]. To test for outliers in our data, we calculated Cook's distance for all observations. Since all values are smaller than 0.5, we do not assume any problematic effects of outliers in our analyses [5].

S1.4. Similarity based on titles and abstracts

The similarity between two papers based on their titles and abstracts was calculated based on the term frequency inverse document frequency (*tf-idf*) of the words (terms) occurring in the abstracts and titles (documents). The *tf-idf* is a standard approach to obtain vector representations for documents. They indicate the relevance of each document's word in the collection of all documents [6]. We used the R package *text2vec* for calculating the *tf-idf*. For each paper, title and abstract were simply concatenated and the list of English stop-words provided by the R package *stopwords* was excluded. Stop-words are the most common words in the English language, such

as “the,” “is,” “which” etc. These words do not add substantial meaning to a text and therefore should be filtered out before text mining procedures. Furthermore, we excluded very infrequent and very frequent words to remove noise. If a word occurs less than three times over all documents, or if the proportion of documents including the word is larger than 0.3, we excluded the word. The *tf-idf* for a word *t* occurring in a document *d* of the document set *D* is defined as follows:

$$tf\text{-}idf(t,d,D) = tf(t,d) \cdot idf(d,D)$$

with

$$tf(t,d) = \frac{number\ of\ occurences\ of\ t\ in\ d}{length\ of\ d}$$

$$idf(d,D) = \log(\frac{number\ of\ documents\ in\ D}{number\ of\ Documents\ in\ D\ containing\ t})$$

tf(t,d) measures how relevant a word *t* is in document *d* (in our case: the abstract and title of a single paper). This is weighted (multiplied) by the rarity of the word in the full set of documents *D* (in our case: the pooled abstracts and titles of all papers included in the analyses). This rarity is measured in the second term by the logarithmized inverse frequency of documents in *D* which contain the word *t*.

To measure the similarity between two papers, the cosine similarity between their *tf-idf* was used. Cut-off values were needed to define different levels of similarity for which we plotted the histograms on the differences in the share of male-authored citing papers between male- and female-authored focal papers. We defined these cut-off values in a way that maximizes the comparability with the approach based on the keywords from the Faculty Opinions database to

define similarity. To achieve this, we set the cut-off values so that the share of pairs that are classified as similar at each level is equal to the share of pairs that are classified as similar at each level when using the Faculty Opinions keywords to define similarity. For instance, at the first level of cosine similarity, 6.5% of pairs are included, because 6.5% of pairs have one shared Faculty Opinions keyword.

S2. Supplementary text

S2.1. Operationalization and results of other studies

Existing studies on gender homophily in citations used different methods and datasets. Table S1 summarizes these studies and shows the main differences with regard to the data and methods used, as well as their results summarized in the gender homophily rate.

Some but not all studies controlled for self-citations by excluding them in their analyses. To operationalize the authors' gender on the paper level, different methods were applied: while some studies considered all authors, others only considered the first author, the first *X* authors, and/or the corresponding authors. Mcelhinny et al. (2003) did not operationalize the authors' gender on the paper level, but analyzed all links between authors and citing authors. The gender homophily rate was generally calculated as the difference in the share of female-authored cited references between female-authored focal papers and male-authored focal papers (for all studies listed in Table S1 the homophily measurement was based on the gender distribution in cited references instead of the citing papers, as we did in our main analyses). To achieve this measurement, the studies did not calculate the share of female-authored (or male-authored) cited references separately for each focal paper to summarize these shares in a next step, but instead pooled the cited references of all female-authored focal papers (male-authored focal papers). The gender homophily rate reported in Table S1 was then calculated as the share of female-authored

cited references of female-authored papers minus the share of female-authored cited references of male-authored papers. Note that for studies that considered all authors of a paper to operationalize the gender on the paper level, mixed author teams neither belonged to the female-authored nor male-authored papers but were instead dropped, as was the case in our analyses based on pairing of papers. Fig. S2 shows the gender homophily rate for all studies listed in Table S1.

All studies restricted their data to a limited number of fields by including only particular journals and publication years, with the only exception being Ghiasi et al. (2018) who did not restrict their analyses to any particular field. Some studies used journals or journal sets also for further controlling the papers' research topics. Ghiasi et al. (2018) additionally used the focal papers' titles and abstracts by matching each female-authored paper to the most similar male-authored paper in the same issue of the same journal (using the gender of the first and corresponding authors to operationalize gender on the paper level). Although this approach considers more information to control for the papers' research and subject area than the approaches used in other studies, the ability to identify papers that are similar in their research questions/topics may be limited due to the relatively small number of papers – and thus topics – covered in a journal issue. Their approach also does not make it possible to control the similarity between papers at different levels of granularity, as we did in our analyses.

S2.2. Robustness checks

In order to test the reliability of our results, we performed several robustness checks. The goal of these additional analyses was to test how the results change when different methodological approaches are applied. In general, the robustness checks support our conclusion: only a small degree of gender homophily in citations can be found after controlling for the similarity between papers, while inadequate measures of topic similarity can inflate the observed gender homophily.

If not explicitly stated otherwise, all analyses described in this section are based on Faculty Opinions data.

S2.2.1. Regression analyses using pairs of focal papers

Pairing the papers for the analyses shown in Fig. 4 allowed us to control the papers' topic at different levels of granularity. When plotting histograms as shown in Fig. 4, only research topics can be controlled by matching the papers at different levels of similarity. However, pairs of focal papers (instead of focal papers themselves, as in the regression analyses shown in the main text and described in S1.3) can also be used to perform regression analyses. In these analyses, all pairs are included where one paper is authored only by male scientists and the other paper is authored only by female scientists. The difference in the share of male-authored citing papers is the dependent variable, and dummy variables for the number of shared Faculty Opinions keywords are the main predictors of interest. Multiple regression analyses make it possible to control not only for research topics, but also for other variables. We controlled in the regression analyses for differences in the quality ratings provided in the Faculty Opinions data (ratings of research quality provided by experts), age (publication year), and team size (number of authors) between both papers of a pair. The three variables are also included in the regression analyses on the level of focal papers presented in Fig. 2. Table S2 and Fig. S3 show the results of the regression analysis based on pairs of focal papers. The model includes dummy variables representing the number of shared Faculty Opinions keywords (one to four or at least five shared Faculty Opinions keywords) and the difference in the Faculty Opinions quality rating, age, and team size for a pair as independent variables. The estimated coefficients for the variables are listed in Table S2. Fig. S3 reveals the predicted difference in the share of male-authored citing papers for the different numbers of shared Faculty Opinions keywords while setting the other variables (difference in

quality ratings, age, and team size) to zero. Setting to zero means that the difference in the share of male-authored citing papers is predicted for the case that both papers of a pair have the same quality rating, age, and team size. The results indicate a pattern similar to the histograms in Fig. 4: the more Faculty Opinions keywords the paired papers share (i.e., the higher their similarity in research topics), the smaller the difference in the share of male-authored citing papers gets. This confirms the result that the gendered citation patterns found in the Faculty Opinions data (in the sense that overall, male scientists are more likely to cite male-authored papers than female scientists, and vice versa) can in large part be explained by the specialization of male and female scientists in different research topics, but not by differences in quality, age or team size. However, a high granularity of topic similarity measurement is needed (based on combinations of keywords) in order to identify the large impact of this structural aspect explaining gendered citation patterns.

S2.2.2. Including self-citations

For the analyses described in the main text, we excluded all self-citations because self-citations artificially increase the observed degree of gender homophily. We defined self-citations as focal paper-citing paper pairs for which at least one author of the focal paper is identical to at least one author of the citing paper [7]. This is based on the assumption that papers were written by the identical author if the focal paper and the citing paper have at least one common author name, taking into account the first initial and the full surname. It can be expected that in some cases the first initial and the full surname are shared by different persons and therefore the assumption that the authors are the same person is wrong. At the same time, it can be assumed that the name representation rarely differs for a person [8]. Thus, this approach for identifying self-citations is likely to overestimate the existence of self-citations, but most self-citations can be assumed to be found.

Fig. S4 shows the histograms for the differences in the share of male-authored citing papers for pairs of focal papers (similar to Fig. 4) when including self-citations. Confirming other studies [e.g., 9, 10, 11], these results show that self-citations contribute to gendered citation patterns. The pattern that the difference in the share of male-authored citing papers becomes smaller the better the topic is controlled (i.e., the minimum number of shared keywords increases) does not change when self-citations are included in the analyses. At the same time, these extended analyses suggest that evidence for gender homophily is overestimated when self-citations are not excluded (as was done in some previous studies, see Table S1). In order to validly measure gender homophily as a preference for citing colleagues of the same gender (and not just one's own work), our empirical evidence clearly reveals that this methodological step of excluding self-citations is very important. There is a much larger average difference in the share of citing papers authored by males left that one might erroneously interpret as gender homophily when not excluding self-citations: the difference increases by 7.2 percentage points when matching papers with at least five shared Faculty Opinions keywords (the difference is then 8.83, instead of 1.61 when excluding self-citations; see Fig. 4). The lager difference that is driven by self-citations should not be confused with homophily.

S2.2.3. Using cited references instead of citing papers

Most of the existing studies on gender homophily in citations analyzed references cited in focal papers in order to answer the question whether female-authored focal papers are less likely to refer to male-authored papers compared to male-authored focal papers. Rather than following this approach of analyzing the cited references in focal papers, we decided to use the papers that cited the focal papers in order to analyze the question whether male-authored focal papers are more likely to be cited by male scientists than female-authored focal papers. The major advantage of

using citing papers (instead of cited references) is that the publication year of the papers (which is used to measure homophily bias) can be held constant to a greater extent in the analyses. The approach allows for a better standardization of the overall gender composition in science, which might influence gender-specific citation patterns. The gender distribution changed over time, and this time-trend may lead to an overestimation of homophily bias: the analysis of cited references can go a long way back in time when the share of female scientists who could be cited was smaller. Older papers written predominantly by men are more likely to be cited by men, simply because they are on average more senior. Senior researchers may work on more classical topics with references reaching longer back in time than junior researchers. Since the focal papers we studied were published no earlier than 2002, the papers citing them could also not have been published before 2002 either. This means that the risk pool of citing papers covers a much smaller time frame than in the analysis of cited references. However, to produce results that are better comparable with most other studies on gender homophily in citations, we considered cited references instead of citing papers in a robustness check. Fig. S5 shows the histograms for the differences in the share of male-authored cited references for pairs of focal papers. Similar to the results shown in Fig. 4, the average difference in the share of male-authored cited references (our indicator for homophily) decreases when topic similarity is controlled for more thoroughly. But when analyzing cited references instead of citing papers, the difference does not diminish as much: in the analysis of cited references, the difference in the share of papers authored by males decreases from 13.76 to 5.23 percentage points, while the difference in the share of male-authored citing papers (the approach we took for our main analysis) diminishes from 12.64 to 1.61 percentage points (see Fig. 4). The difference between both results suggests that controlling publication year in the analysis is important in order to validly measure gender homophily in citations. In the analysis of citing

papers, the publication year of the papers can be hold constant to a greater extent than in the analysis of cited references (see above).

S2.2.4. Gender assignments

For a given first name and country (if available), the database that we used for inferring the authors' gender differentiates between "is mostly female/male" and "is female/male". In our main analyses, we used both types of gender classification. Fig. S6 presents empirical results as shown in Fig. 4 but dropping all names with less reliable gender classifications: in the histograms, the difference in the share of male-authored citing papers for pairs of focal papers is shown only for papers with the more reliable gender assignment "is female/male." The results are very similar to using both types of gender classification: the difference in the share of male-authored citing papers decreases from 13.58 to 0.62 percentage points when using the more restrictive gender assignment, while it diminishes from 12.64 to 1.61 percentage points when using the less restrictive assignments including also the "mostly female/male" classification (see again Fig. 4 in our main analyses). We conclude that the reliability of gender assignments indicated by the database does not play a significant role for our results.

S2.2.5. Alternative approaches for controlling similarity

Similarity between papers can be controlled by various approaches. We deem abstracts and titles the most adequate alternative to Faculty Opinions keywords (when expanding the results to papers not included in the Faculty Opinions database). Abstracts and titles usually contain comprehensive information about the content and research topics of papers. Fig. 5B and Fig. 5F in the main text show average differences in the share of male-authored citing papers when using abstracts and titles to match the papers. Fig. S7-Fig. S8 present the corresponding histograms based on this alternative approach for both Faculty Opinions and WoS data. Besides using titles and

abstracts, other possibilities for controlling similarity between papers are the number of shared cited references, the number of shared keywords provided in the WoS, or the number of shared WoS subject categories [12]. WoS keywords include both keywords specified by a paper's authors and keywords automatically generated based on the titles of a paper's cited references [13, 14]. Fig. S9-Fig. S11 show the results for these other approaches using the Faculty Opinions data. When interpreting the results in the figures, it should be considered that the similarity levels do not align with the similarity levels based on the Faculty Opinions keywords; i.e., there is a lower or higher number of paper pairs that are categorized into a certain similarity level than when using the keyword approach. This is because the number of shared cited references, WoS keywords, and WoS subject categories are discrete values: such values cannot be perfectly recategorised into different similarity levels (shares of papers to be found to be similar) that result when using different numbers of Faculty Opinions keywords to define similarity levels. Replication of the percentage distribution across different similarity categories based on Faculty Opinions keywords is only possible with the measurement of similarity based on abstracts and titles. The cosine similarity is a continuous measure that can be categorized at arbitrary cut-off values.

Although we used very different approaches for measuring paper similarity, we always found the same pattern: the better the topic similarity between papers is controlled for, the smaller the difference in the share of male-authored citing papers gets. Since the approaches differ with regard to the share of paper pairs that fall into different similarity levels, the approaches cannot be directly compared. However, one might argue that different similarity levels of two approaches are roughly comparable if their numbers of pairs are approximately equal. For example, the number of pairs with at least one shared cited reference (23,192) roughly corresponds to the number of pairs that share at least four Faculty Opinions keywords (14,167). Thus, comparing these similarity

levels of the two approaches means comparing the 23,192 most similar paper pairs when measuring the similarity based on shared cited references with the 14,167 most similar pairs when measuring the similarity based on shared Faculty Opinions keywords. For this comparison, the difference in the share of male-authored citing papers (our indicator of homophily) replicates well: it is only slightly higher when using the overlap in cited references instead of keywords to define similarity (5.88 vs. 3.48 percentage points; see Fig. S9). Likewise, three shared cited references (resulting in 2,354 pairs that could be matched) roughly correspond to five shared Faculty Opinions keywords (resulting in 3,010 pairs that could be matched). At this level of similarity, the difference in the share of male-authored citing papers is also only slightly higher when using cited references for measuring topic similarity (2.75 vs. 1.61 percentage points).

The effect of controlling WoS keywords is comparable to the effect of controlling similarity based on titles and abstracts for the Faculty Opinions data. The difference in the share of male-authored citing papers decreases from 12.64 percentage points when including all pairs of papers to 7.27 when including the 2,312 pairs of papers with at least three shared WoS keywords (see Fig. S10). For the approach based on abstracts and titles, the difference in the share of male-authored citing papers decreases from 12.76 to 6.41 percentage points at the highest level of similarity, where 2,707 papers could be matched. This decrease is also very similar to the one observed when using at least three shared WoS subject categories (which is from 12.64 to 5.41 percentage points; see Fig. S11). Further restricting the pairs of focal papers to those with at least four or five shared WoS subject categories increases the difference in the share of male-authored citing papers. However, these analyses are based only on a very small number of cases (126 and 44 pairs of focal papers). A small number of cases implies a limited reliability of the results.

All in all, our extensions to other measures of similarity indicate a strong robustness of our main conclusion: topic similarity is an important structural aspect that should be controlled when one is interested in direct gender effects, such as gender homophily bias. Only when the specialization of male and female scientists on research topics is controlled for with exact measurements of the different research topics and questions they work on, one can see the “bias” that leads same gender peers to cite each other more often than what a baseline model of gender-blind selection of relevant literature would predict. A noteworthy side-result of our extensions to other measures is their evaluation in regard to their reliability in capturing topic similarity. It appears that expert ratings based on standardized lists of keywords are more suitable in defining risk pools of papers that belong to a common research field/topic than measurements based on alternative indicators of papers’ content. Similarity measures based on abstracts or titles, cited references or WoS keywords may offer viable alternative measures of topic similarity when expert ratings are not available (as in the Faculty Opinions data). However, their disadvantage is that only a small number of pairs with (several) shared cited references or WoS keywords can be matched. Titles and abstracts allow for a more nuanced measurement of topic similarity, since they allow defining different levels of similarity with a considerable number of pairs that can be matched. The key take-away of these robustness checks is, however, that thorough controls for research topics are important for identifying genuine gender effects.

S2.2.6. Excluding papers with extreme gender distributions among citing papers

Papers included in the Faculty Opinions data are on average cited more often than other papers in the same field [15]. Thus, there are more focal papers with large citation counts in the Faculty Opinions data (that we used in the main analyses in this study) than in the WoS data. Having smaller citation counts in the WoS data increases the chance of having 100% female-

authored or 100% male-authored citing papers, because there are fewer possible shares of female-authored and male-authored citing papers. For example, a paper with one citation can only have 0% or 100% male-authored (female-authored) citing papers, a paper with two citations can only have 0%, 50% or 100% male-authored (female-authored) citing papers (again, excluding mixed-authored papers, to be able to pair only male- and female-authored papers). While papers with large citation counts may have a share of male-authored (female-authored) citing papers close to but less than 100%, most papers with few citation counts would have a share of male-authored (female-authored) citing papers of exactly 100% in a similar situation. For the pairs of focal papers, this increases the chance of having no difference in the share of male-authored citing papers (which may be a reason for the large number of pairs with a difference of zero in Fig. S8). However, if there is a gender homophily bias in citation decisions, the average difference in the share of male-authored citing papers on the aggregated level should still be larger than zero for the focal papers with only one (a few) citation(s). In order to test whether the large share of pairs of focal papers with no difference in the share of male-authored citing papers affects our results, we generated the histograms for the pairs of focal papers after excluding all papers with 100% female-authored or 100% male-authored citing papers. We did this for the Faculty Opinions and WoS data, using abstracts and titles for measuring the similarity between papers in both cases. In either case, including and excluding papers with only female-authored or only male-authored citing papers produces almost identical results. The share of male-authored citing papers reduces from 12.76 to 6.41 percentage points (Faculty Opinions data; see Fig. S7) and from 8.68 to 3.63 percentage points (WoS data; see Fig. S8) when including papers with extreme gender distributions among citing papers (100% male-authored or female-authored papers). When excluding them, the difference in the share of male-authored citing papers decreases from 12.99 to 6.51 percentage points (Faculty

Opinions data; see Fig. S12) and from 9.70 to 4.02 percentage points (WoS data; see Fig. S13). Thus, we observe the same pattern as in other analyses: controlling for the similarity between papers reduces the difference in the share of male-authored citing papers as evidence for gender homophily; and this result is very robust to using alternative sample restrictions (here: including and excluding papers with extreme gender distributions among citing papers).

S2.2.7. Level of analysis

Using pairs of focal papers may result in certain papers having a stronger influence on the results than other papers. For example, imagine two female-authored papers: paper A, for which there are nine male-authored papers with two shared Faculty Opinions keywords, and paper B, for which there is only one male-authored paper with two shared Faculty Opinions keywords. This means that in the analysis based on all possible pairs with at least two shared Faculty Opinions keywords, nine pairs containing paper A are considered, but only one pair containing paper B. In this scenario, paper A would have a stronger influence on the result than paper B, since 90% of pairs of papers are based on paper A and only 10% on paper B. Extreme values in the share of male-authored citing papers for papers that are included in many pairs would have a great effect on the results. We assume that this should not make a difference, since extreme values can be expected to occur at both ends of the spectrum between a small and a large share of male-authored citing papers. In order to empirically verify our assumption, we performed additional analyses by changing the level of analysis from pairs of focal papers to focal papers. For every male-authored focal paper, we calculated the average difference in the share of male-authored citing papers to all paired female-authored focal papers. This results in one value for each male-authored focal paper, which can be interpreted as the average difference in the share of male-authored citing papers between the male-authored focal paper and its paired female-authored focal papers.

Fig. S14 shows the histograms of these values by the number of shared Faculty Opinions keywords. Fig. S15 shows the corresponding results when aggregating the differences in the share of male-authored citing papers for each female-authored focal paper instead of each male-authored focal paper. The results of these two analyses differ only slightly from each other: the difference in the share of male-authored citing papers decreases from 12.64 to 2.70 percentage points when aggregating over male-authored focal papers and from 12.64 to 3.77 percentage points when aggregating over female-authored focal papers. Both results also differ only slightly from the results obtained when not aggregating over focal papers (recall again the result from our main analysis, in which the difference in the share of male-authored citing papers decreases from 12.64 to 1.61 percentage points). This makes us confident that the findings of our main approach of studying pairs of focal papers is not driven by some dominant "outlier" papers that have particularly strong influence on the results.

S2.2.8. Share of female-authored citing papers instead of male-authored citing papers

In our main analyses, we focused on the share of male-authored citing papers in order to assess the degree of gender homophily in citation decisions. Since gender homophily in citations is the preference to cite authors of the same gender, gender homophily can also be operationalized by the difference in the share of female-authored citing papers. If female authors were more likely to cite other female authors, the share of female-authored citing papers would differ between male-authored and female-authored focal papers. Fig. S16 shows the histograms for the differences in the share of female-authored citing papers for all pairs of focal papers from the Faculty Opinions data using Faculty Opinions keywords to control for paper similarity. Here, we calculated the differences as the share of female-authored citing papers for the male-authored focal paper minus

the share for the female-authored focal paper. This means that negative values indicate gender homophily in citations.

Without controlling for the similarity between papers, the difference between male-authored and female-authored focal papers in the share of female-authored citing papers is smaller than in the share of male-authored citing papers (8.21 vs. 12.64 percentage points). This may be due to the generally small share of female-authored citing papers: if both papers of a pair have a relatively small share of female-authored citing papers, the difference between them cannot be large either. In line with the analyses using the share of male-authored citing papers, the difference in the share of female-authored citing papers decreases when controlling for the similarity between papers. Most of the gender differences in citations disappears after controlling for the similarity between papers, and there is only a small degree of gender homophily in citations left once topic similarity is controlled. The remaining absolute difference is 2.4 percentage points, which is close to the 1.6 percentage points when using male-authored citing papers (see Fig. 4). The only major contrast to the share of male-authored citing papers is as follows: when using the share of female-authored (instead of male-authored) citing papers, already the first levels of topic similarity based on only one or two shared Faculty Opinions keywords are sufficient to net out nearly all gender differences. The difference hardly shrinks further when controlling for more than two Faculty Opinions keywords. One reason for this result may be the smaller average difference in the share of female-authored citing papers when not controlling for similarity: if there is only a small average difference, it cannot get much smaller any more when (further) controlling for the similarity.

These robustness checks (based on the difference in the share of female-authored citing papers) also support our main finding that controlling for the similarity between papers is

important, even though the granularity of this similarity is not as important as when using the share of male-authored citing papers.

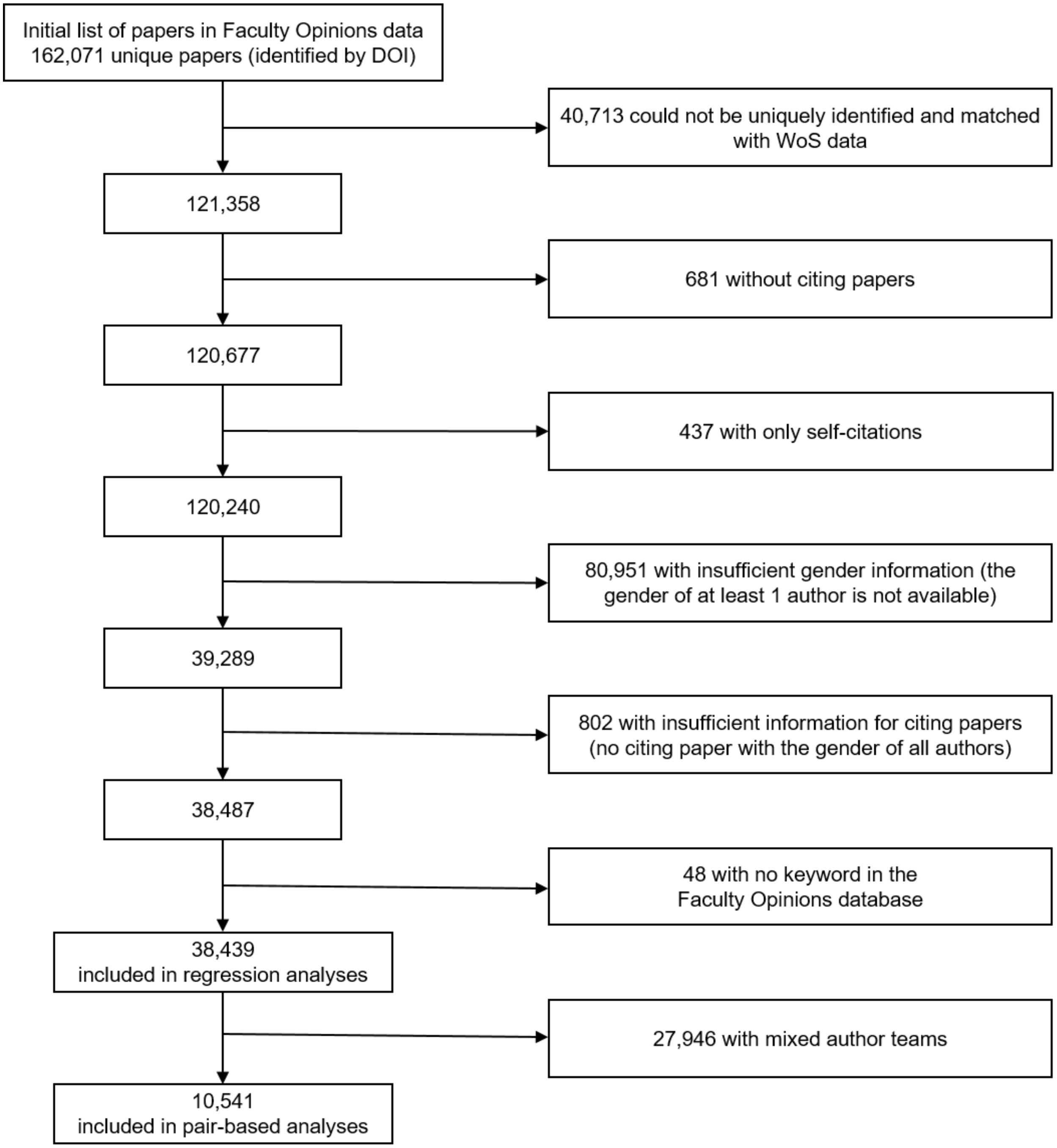

Fig. S1. Number of papers included in the main analyses.

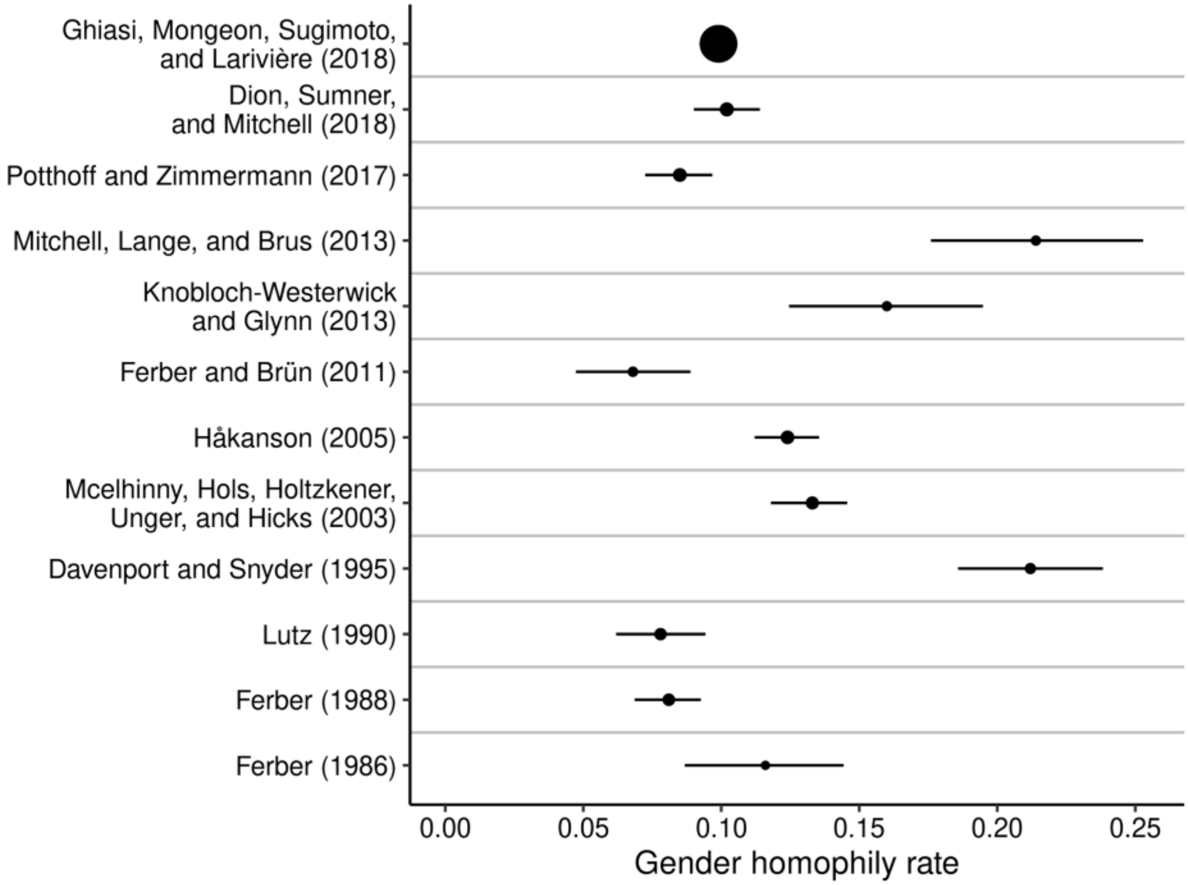

Fig. S2. Gender homophily rates for previous studies. Symbol sizes are proportional to the square root of the sample size. The lines show 95% confidence intervals. Studies are ordered chronologically. See S2.1 and Table S11 for details on the calculation of the gender homophily rates.

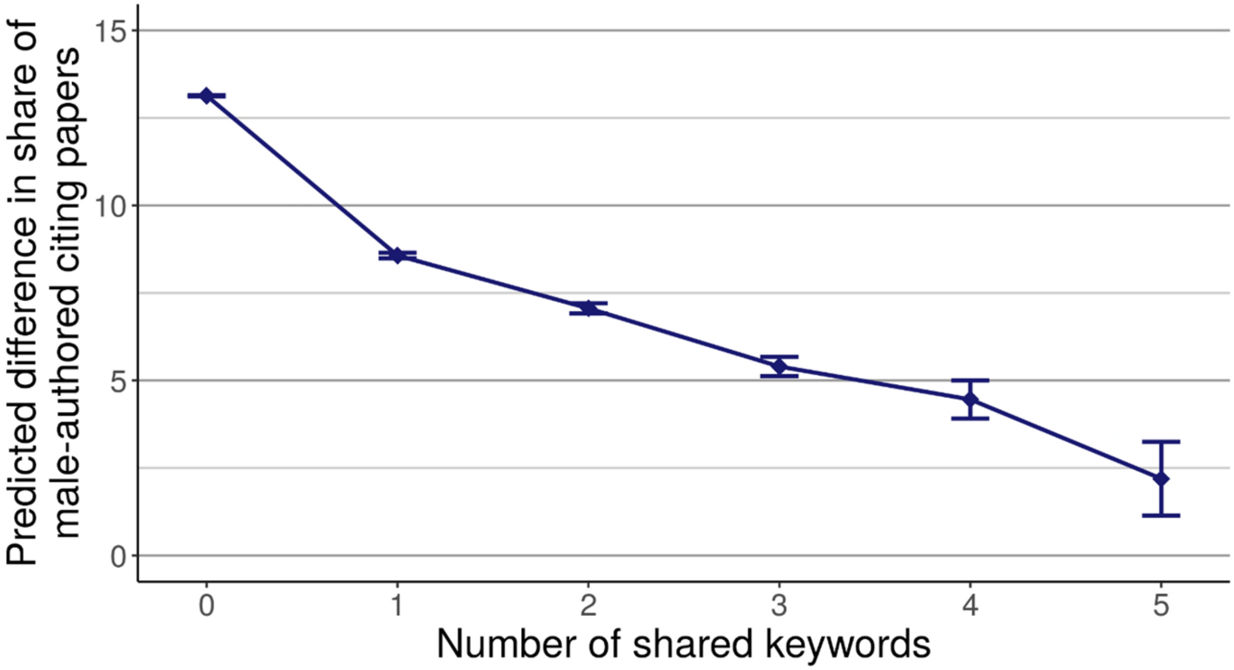

Fig. S3. Marginal effects of the number of shared Faculty Opinions keywords. The marginal effects are calculated based on the regression results presented in Table S2 for pairs of focal papers using the Faculty Opinions data. The dependent variable is the difference in the share of male-authored citing papers. Other predictor variables (difference in quality ratings, age, and team size) are set to zero.

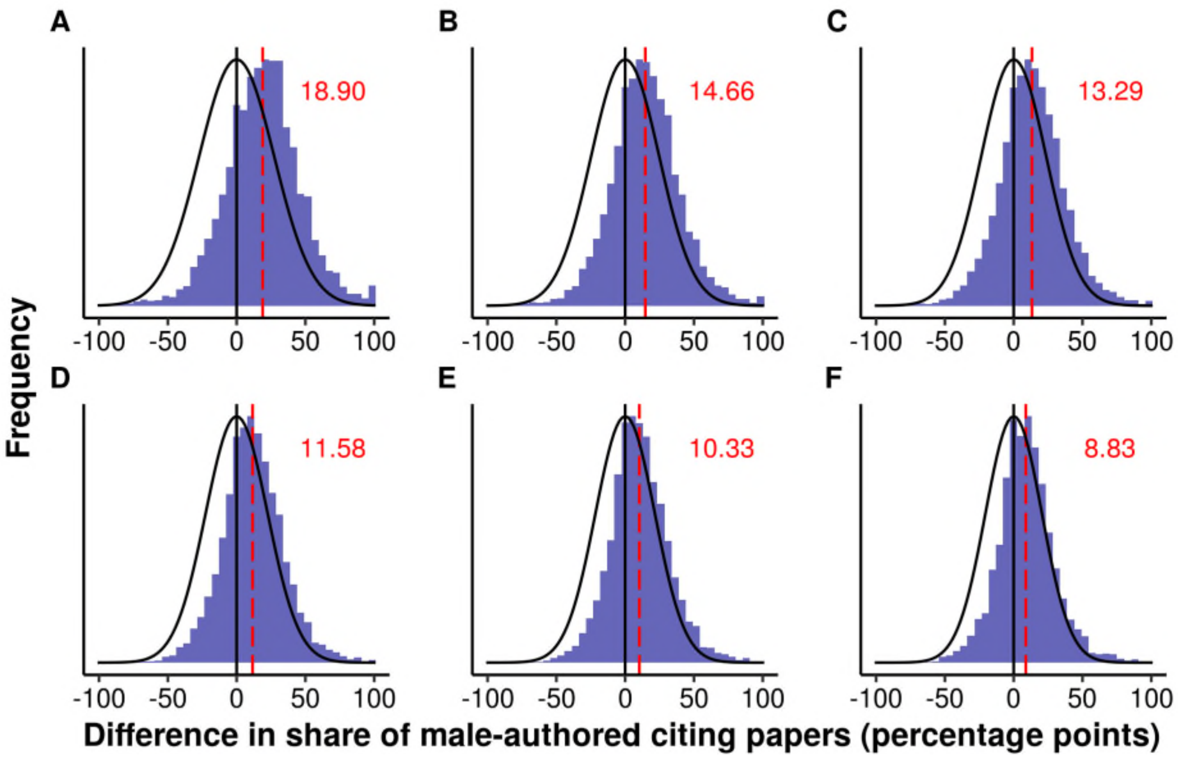

Fig. S4. Histograms for the differences in the share of male-authored citing papers for pairs of focal papers (Faculty Opinions data, including self-citations). In each histogram, the pairs of focal papers are restricted to those cases in which one focal paper is authored only by male scientists and the other focal paper is authored only by female scientists. Positive differences result when the male-authored paper of a pair has a higher share of male-authored citations than the female-authored paper of this pair. The histograms differ in the minimum number of shared Faculty Opinions keywords that the pairs of focal papers have, and – as a consequence – in the number of pairs of focal papers included: all 11,932,148 pairs in (A), 779,244 pairs with at least one shared Faculty Opinions keyword in (B), 227,093 pairs with at least two shared Faculty Opinions keywords in (C), 59,122 pairs with at least three shared Faculty Opinions keywords in (D), 14,305 pairs with at least four shared Faculty Opinions keywords in (E), and 3,035 pairs with at least five shared Faculty Opinions keywords in (F). The vertical lines are placed at 0 (black) and at the observed average difference (red, dashed). The black curve shows the shape of a normal distribution.

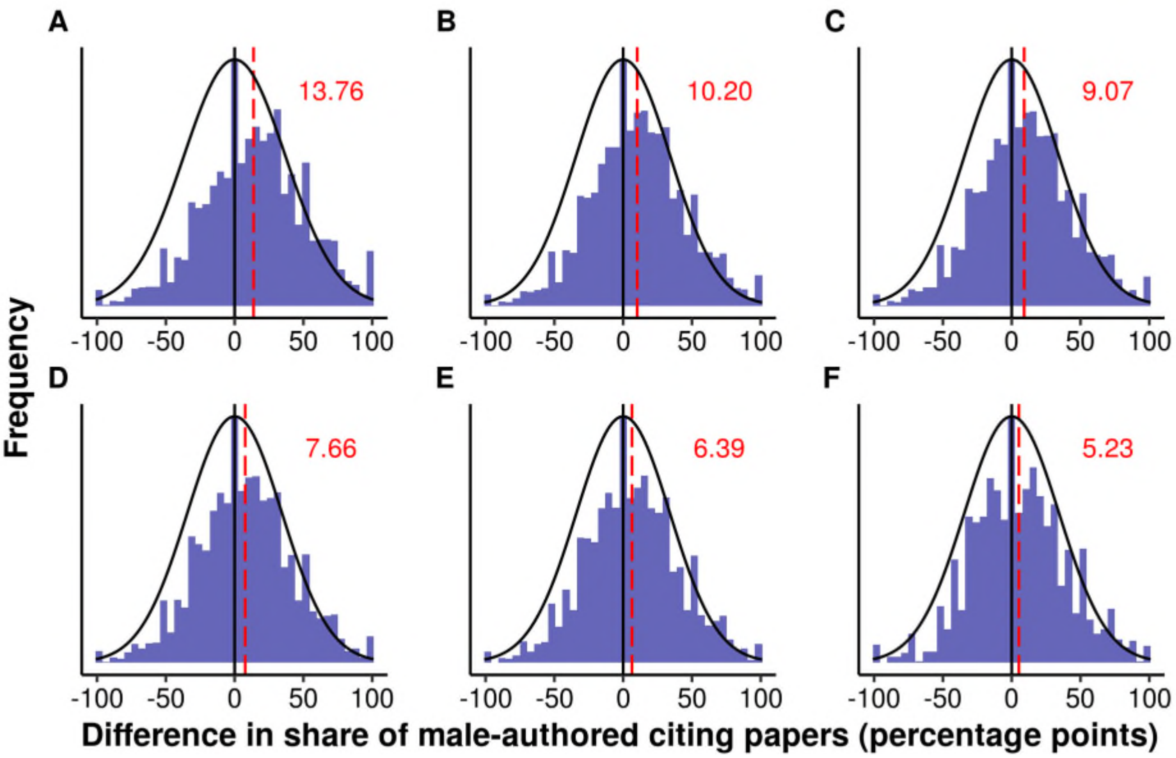

Fig. S5. Histograms for the differences in the share of male-authored cited references for pairs of focal papers (Faculty Opinions data). In each histogram, the pairs of focal papers are restricted to those cases in which one focal paper is authored only by male scientists and the other focal paper is authored only by female scientists. Positive differences result when the male-authored paper of a pair has a higher share of male-authored cited references than the female-authored paper of this pair. The histograms differ in the minimum number of shared Faculty Opinions keywords that the pairs of focal papers have, and – as a consequence – in the number of pairs of focal papers included: all 9,654,680 pairs in (A), 633,651 pairs with at least one shared Faculty Opinions keyword in (B), 182,092 pairs with at least two shared Faculty Opinions keywords in (C), 46,830 pairs with at least three shared Faculty Opinions keywords in (D), 11,005 pairs with at least four shared Faculty Opinions keywords in (E), and 2,241 pairs with at least five shared Faculty Opinions keywords in (F). The vertical lines are placed at 0 (black) and at the observed average difference (red, dashed). The black curve shows the shape of a normal distribution.

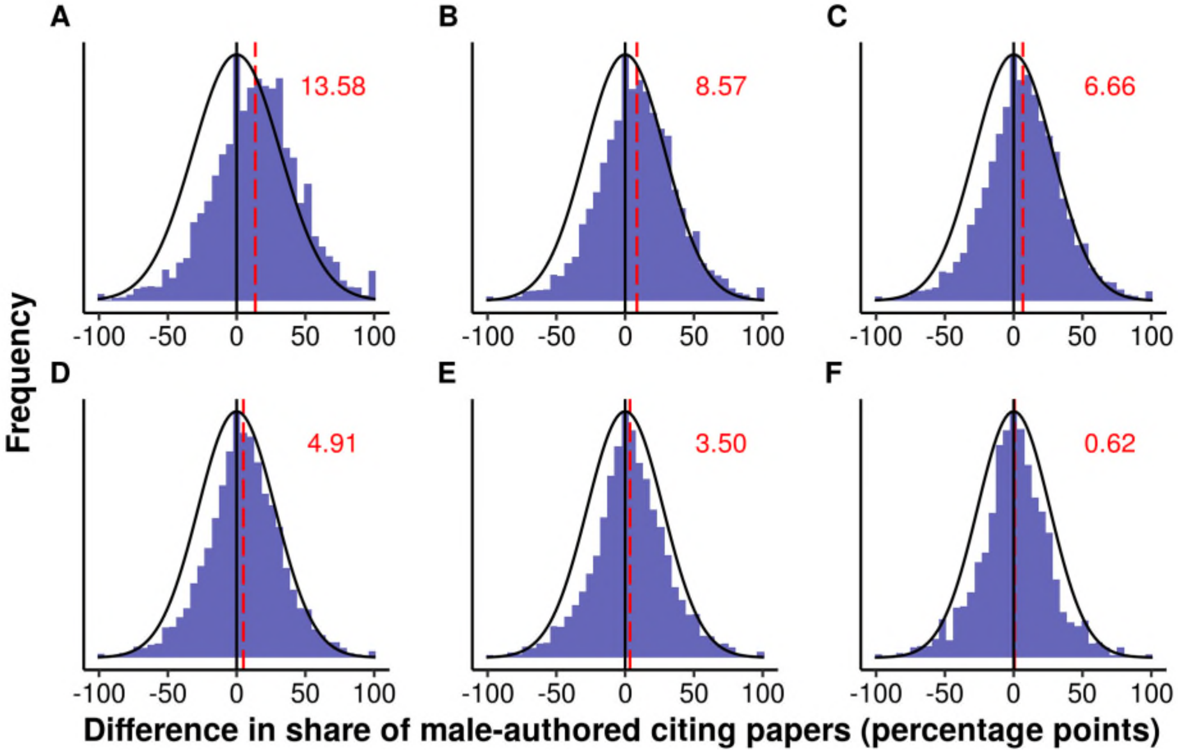

Fig. S6. Histograms for the differences in the share of male-authored citing papers for pairs of focal papers (Faculty Opinions data, based on more restrictive gender assignments). In each histogram, the pairs of focal papers are restricted to those cases in which one focal paper is authored only by male scientists and the other focal paper is authored only by female scientists. Positive differences result when the male-authored paper of a pair has a higher share of male-authored citing papers than the female-authored paper of this pair. The histograms differ in the minimum number of shared Faculty Opinions keywords that the pairs of focal papers have, and – as a consequence – in the number of pairs of focal papers included: all 7,532,670 pairs in (A), 514,725 pairs with at least one shared Faculty Opinions keyword in (B), 150,964 pairs with at least two shared Faculty Opinions keywords in (C), 39,729 pairs with at least three shared Faculty Opinions keywords in (D), 9,818 pairs with at least four shared Faculty Opinions keywords in (E), and 2,107 pairs with at least five shared Faculty Opinions keywords in (F). The vertical lines are placed at 0 (black) and at the observed average difference (red, dashed). The black curve shows the shape of a normal distribution.

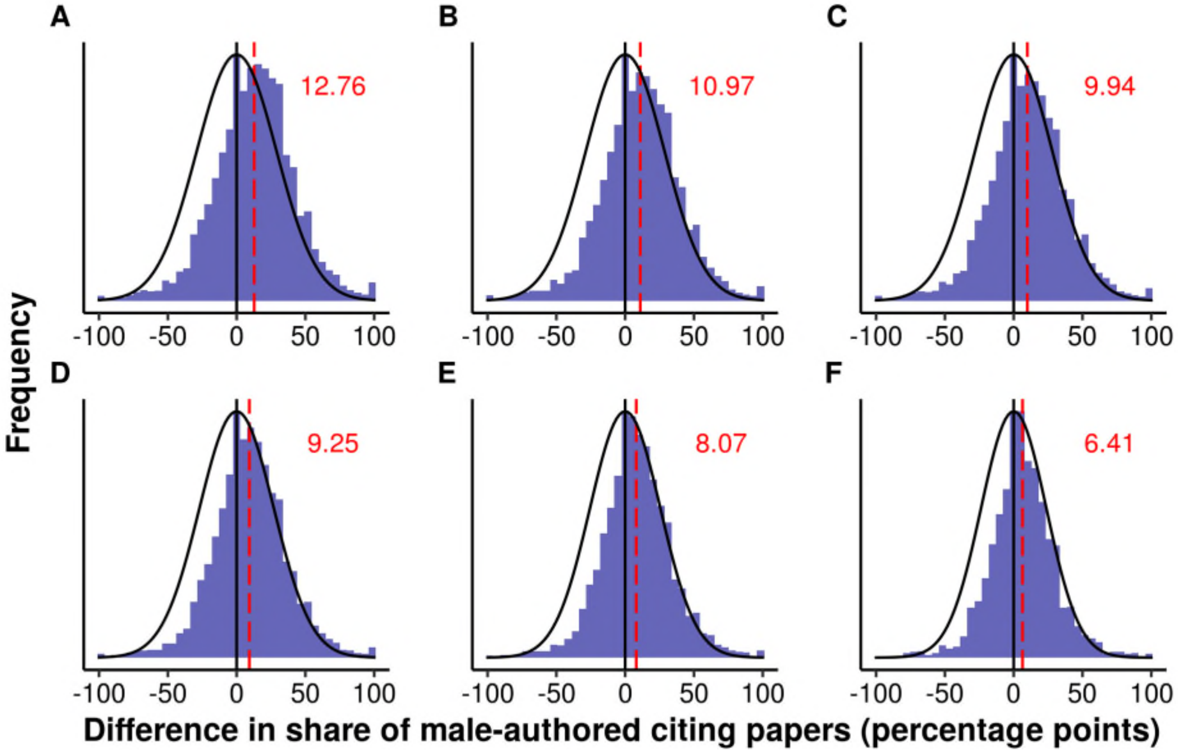

Fig. S7. Histograms for the differences in the share of male-authored citing papers for pairs of focal papers (Faculty Opinions data, using titles and abstracts for measuring the similarity between papers). In each histogram, the pairs of focal papers are restricted to those cases in which one focal paper is authored only by male scientists and the other focal paper is authored only by female scientists. Positive differences result when the male-authored paper of a pair has a higher share of male-authored citing papers than the female-authored paper of this pair. The histograms differ in the minimum cosine similarity between the tf-idf of two paired papers, and – as a consequence – in the number of pairs of focal papers included: all 10,730,525 pairs in (A), 703,162 pairs with a cosine similarity of at least 0.026 in (B), 204,596 pairs with a cosine similarity of at least 0.047 in (C), 53,013 pairs with a cosine similarity of at least 0.084 in (D), 12,746 pairs with a cosine similarity of at least 0.147 in (E), and 2,707 pairs with a cosine similarity of at least 0.246 in (F). The vertical lines are placed at 0 (black) and at the observed average difference (red, dashed). The black curve shows the shape of a normal distribution.

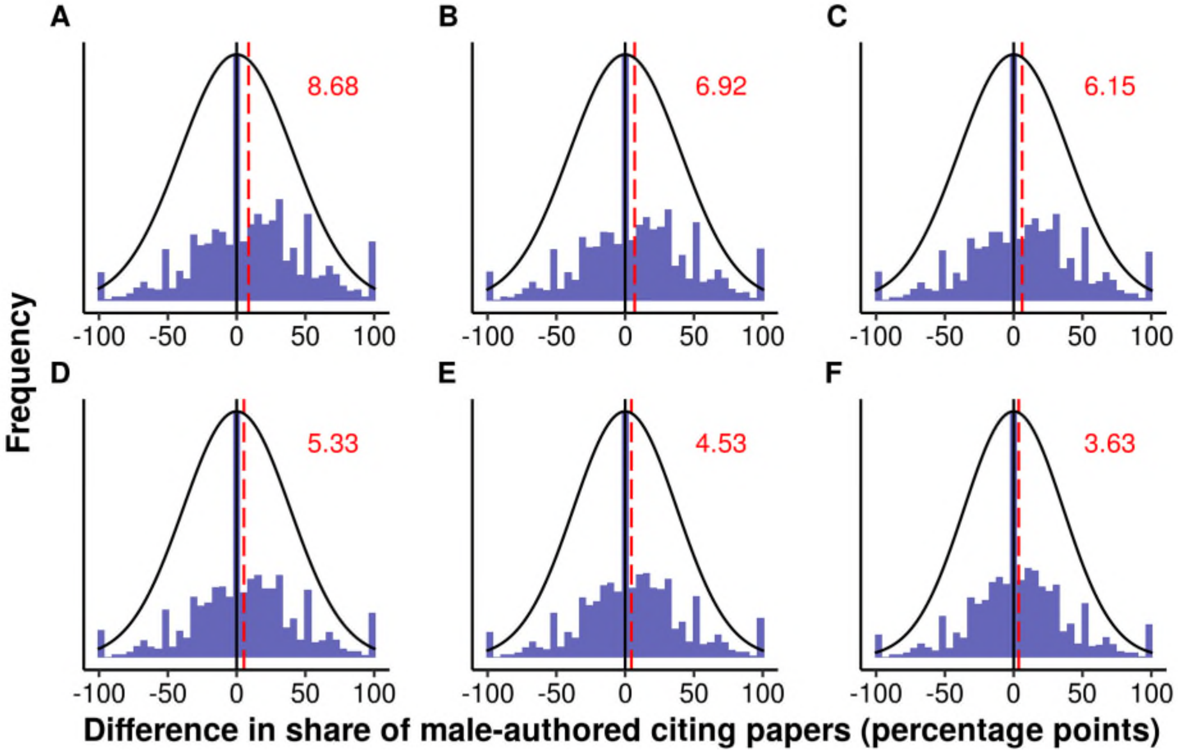

Fig. S8. Histograms for the differences in the share of male-authored citing papers for pairs of focal papers (WoS data, using titles and abstracts for measuring the similarity between papers). In each histogram, the pairs of focal papers are restricted to those cases in which one focal paper is authored only by male scientists and the other focal paper is authored only by female scientists. Positive differences result when the male-authored paper of a pair has a higher share of male-authored citing papers than the female-authored paper of this pair. The histograms differ in the minimum cosine similarity between the tf-idf of two paired papers, and – as a consequence – in the number of pairs of focal papers included: all 508,941,740 pairs in (A), 32,903,466 pairs with a cosine similarity of at least 0.036 in (B), 9,572,570 pairs with a cosine similarity of at least 0.064 in (C), 2,510,050 pairs with a cosine similarity of at least 0.112 in (D), 615,604 pairs with a cosine similarity of at least 0.185 in (E), and 133,381 pairs with a cosine similarity of at least 0.286 in (F). The vertical lines are placed at 0 (black) and at the observed average difference (red, dashed). The black curve shows the shape of a normal distribution.

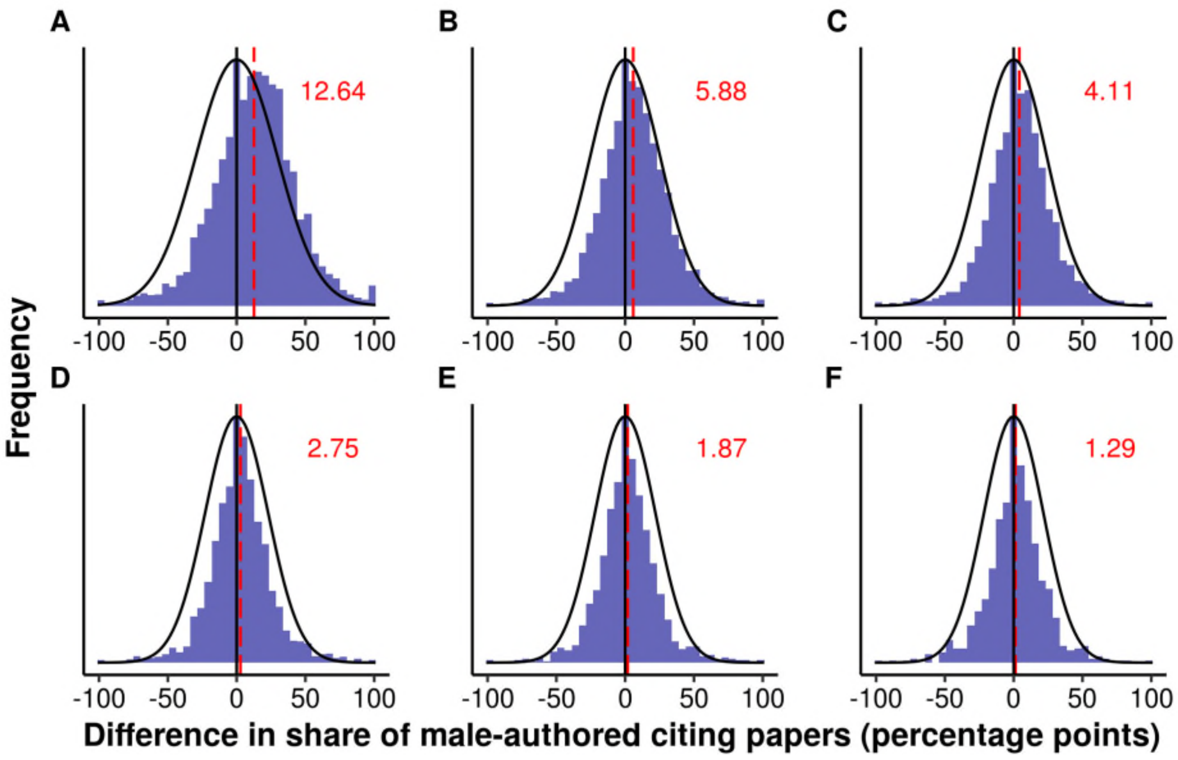

Fig. S9. Histograms for the differences in the share of male-authored citing papers for pairs of focal papers (Faculty Opinions data, using the number of shared cited references for measuring the similarity between papers). In each histogram, the pairs of focal papers are restricted to those cases in which one focal paper is authored only by male scientists and the other focal paper is authored only by female scientists. Positive differences result when the male-authored paper of a pair has a higher share of male-authored citing papers than the female-authored paper of this pair. The histograms differ in the minimum number of shared cited references that the pairs of focal papers have, and – as a consequence – in the number of pairs of focal papers included: all 11,702,080 pairs in (A), 23,192 pairs with at least one shared cited reference in (B), 5,479 pairs with at least two shared cited references in (C), 2,354 pairs with at least three shared cited references in (D), 1,304 pairs with at least four shared cited references in (E), and 798 pairs with at least five shared cited references in (F). The vertical lines are placed at 0 (black) and at the observed average difference (red, dashed). The black curve shows the shape of a normal distribution.

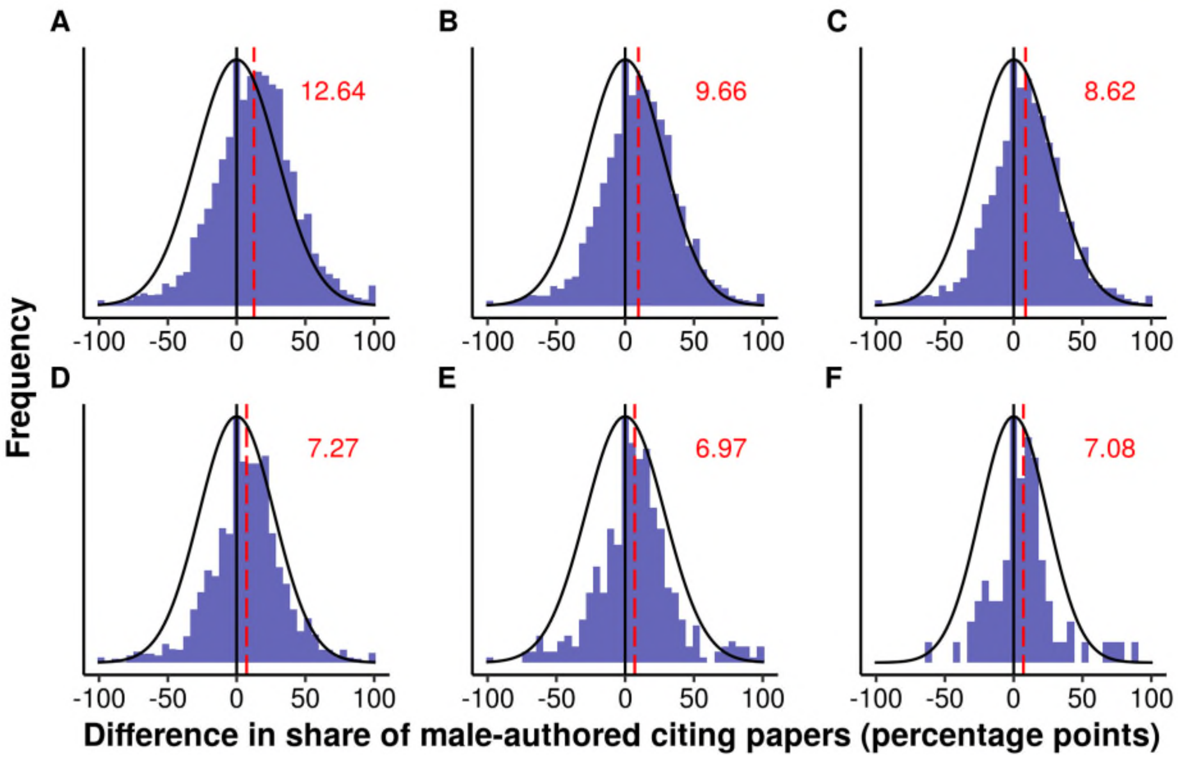

Fig. S10. Histograms for the differences in the share of male-authored citing papers for pairs of focal papers (Faculty Opinions data, using the number of shared WoS keywords for measuring the similarity between papers). In each histogram, the pairs of focal papers are restricted to those cases in which one focal paper is authored only by male scientists and the other focal paper is authored only by female scientists. Positive differences result when the male-authored paper of a pair has a higher share of male-authored citing papers than the female-authored paper of this pair. The histograms differ in the minimum number of shared WoS keywords that the pairs of focal papers have, and – as a consequence – in the number of pairs of focal papers included: all 11,702,080 pairs in (A), 330,072 pairs with at least one shared WoS keyword in (B), 23,074 pairs with at least two shared WoS keywords in (C), 2,312 pairs with at least three shared WoS keywords in (D), 409 pairs with at least four shared WoS keywords in (E), and 81 pairs with at least five shared WoS keywords in (F). The vertical lines are placed at 0 (black) and at the observed average difference (red, dashed). The black curve shows the shape of a normal distribution.

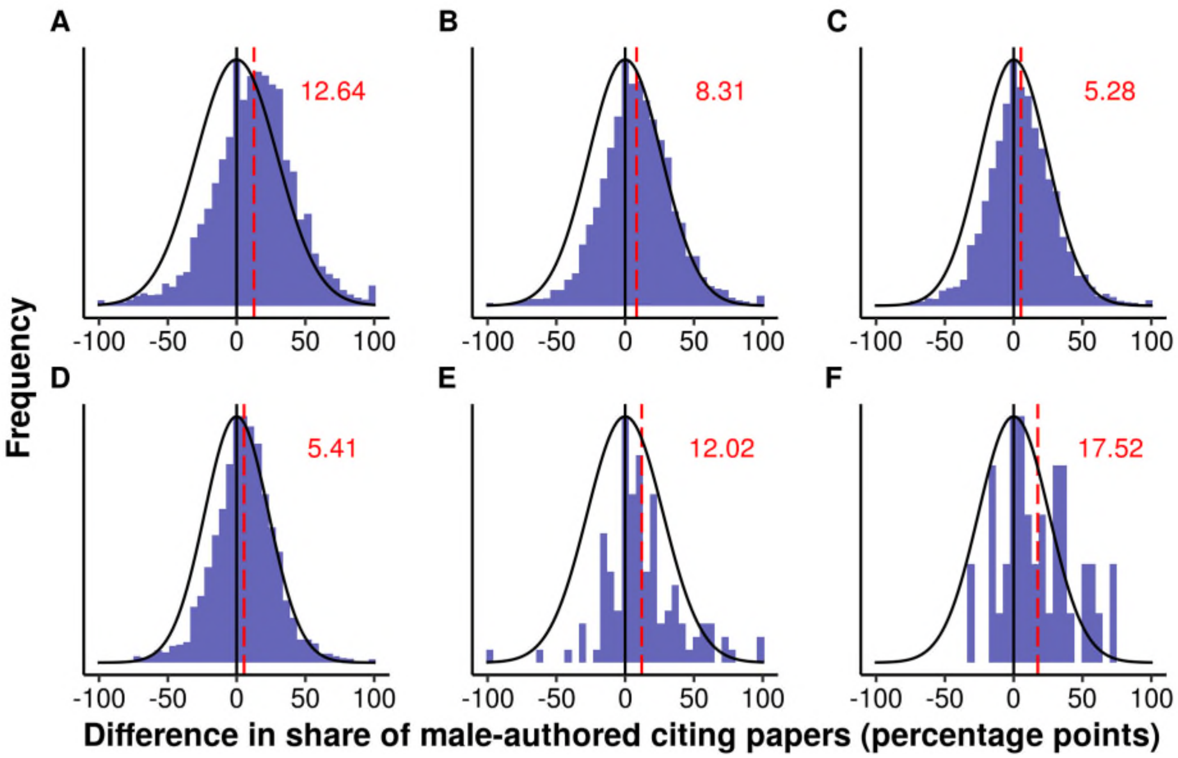

Fig. S11. Histograms for the differences in the share of male-authored citing papers for pairs of focal papers (Faculty Opinions data, using the number of shared WoS subject categories for measuring the similarity between papers). In each histogram, the pairs of focal papers are restricted to those cases in which one focal paper is authored only by male scientists and the other focal paper is authored only by female scientists. Positive differences result when the male-authored paper of a pair has a higher share of male-authored citing papers than the female-authored paper of this pair. The histograms differ in the minimum number of shared subject categories that the pairs of focal papers have, and – as a consequence – in the number of pairs of focal papers included: all 11,702,080 pairs in (A), 745,887 pairs with at least one shared subject category in (B), 56,975 pairs with at least two shared subject categories in (C), 6,180 pairs with at least three shared subject categories in (D), 126 pairs with at least four shared subject categories in (E), and 44 pairs with at least five shared subject categories in (F). The vertical lines are placed at 0 (black) and at the observed average difference (red, dashed). The black curve shows the shape of a normal distribution.

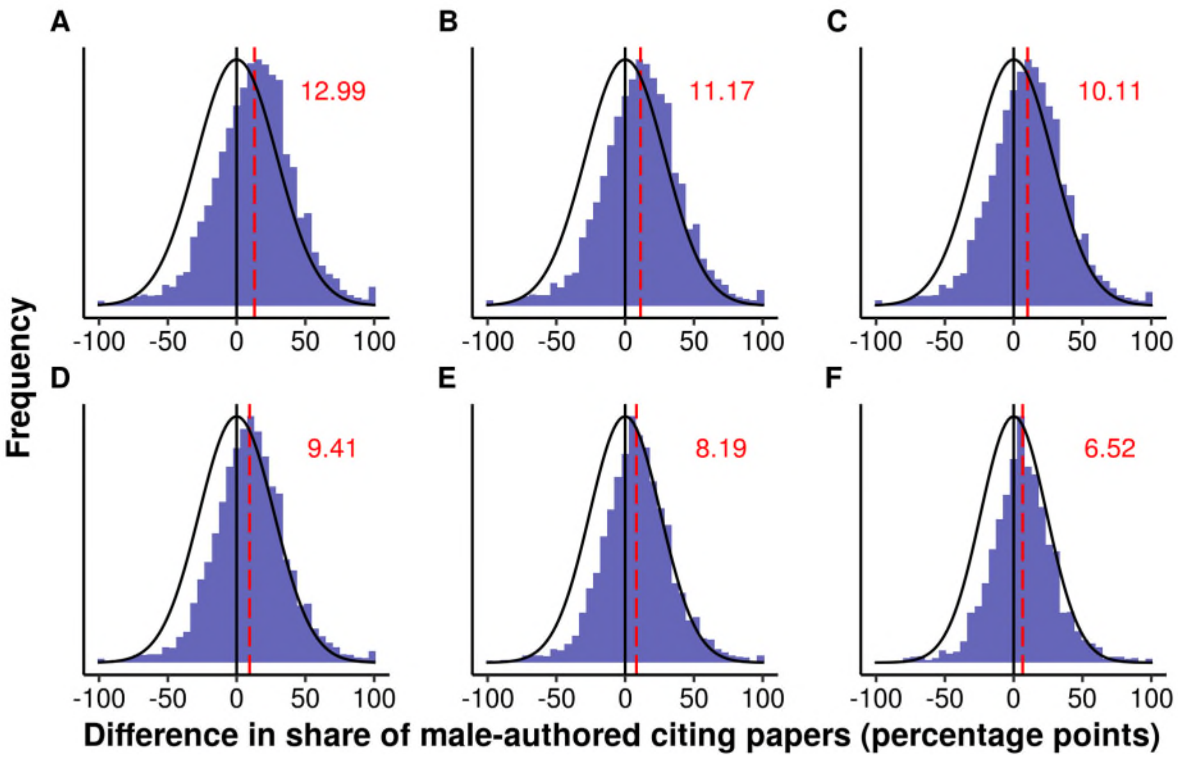

Fig. S12. Histograms for the differences in the share of male-authored citing papers for pairs of focal papers (Faculty Opinions data, using titles and abstracts for measuring paper similarity and excluding papers with extreme gender distributions among citing papers). In each histogram, the pairs of focal papers are restricted to those cases in which one focal paper is authored only by male scientists and the other focal paper is authored only by female scientists. Positive differences result when the male-authored paper of a pair has a higher share of male-authored citing papers than the female-authored paper of this pair. The histograms differ in the minimum cosine similarity between the tf-idf of two paired papers, and – as a consequence – in the number of pairs of focal papers included: all 10,544,969 pairs in (A), 689,035 pairs with a cosine similarity of at least 0.026 in (B), 200,286 pairs with a cosine similarity of at least 0.047 in (C), 51,895 pairs with a cosine similarity of at least 0.084 in (D), 12,486 pairs with a cosine similarity of at least 0.147 in (E), and 2,650 pairs with a cosine similarity of at least 0.246 in (F). The vertical lines are placed at 0 (black) and at the observed average difference (red, dashed). The black curve shows the shape of a normal distribution.

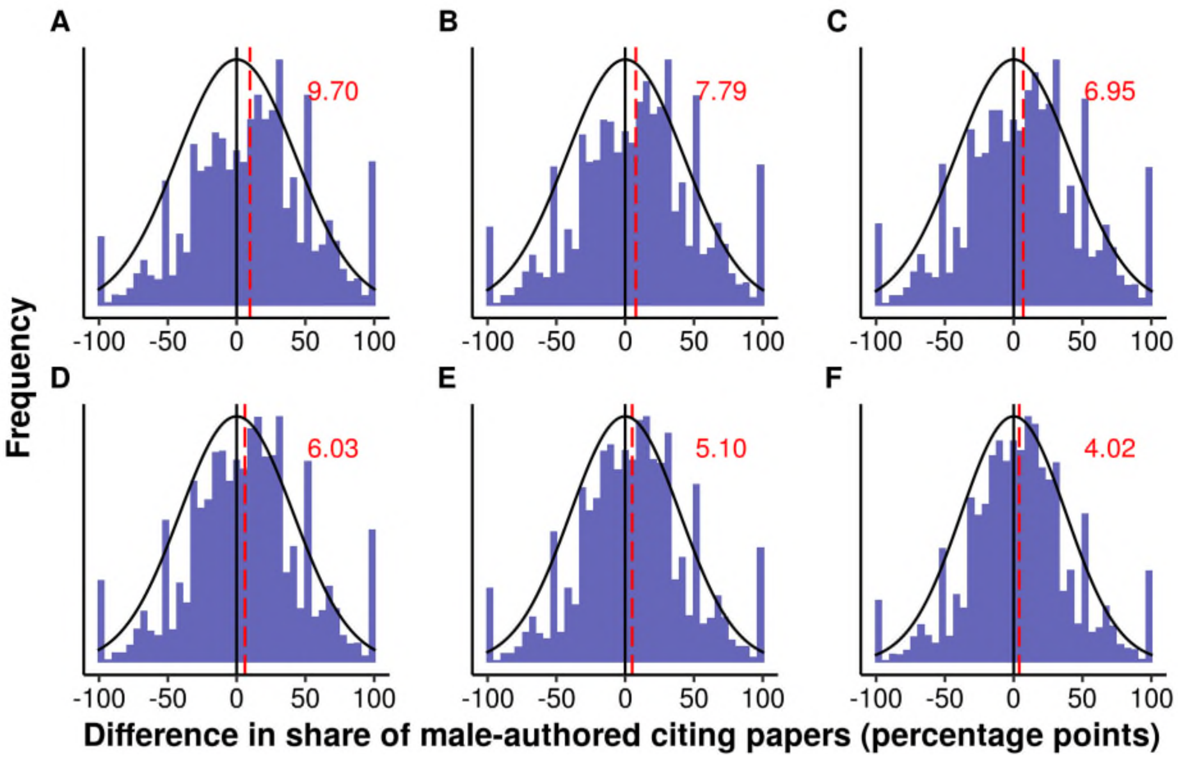

Fig. S13. Histograms for the differences in the share of male-authored citing papers for pairs of focal papers (WoS data, using titles and abstracts for measuring paper similarity and excluding papers with extreme gender distributions among citing papers). In each histogram, the pairs of focal papers are restricted to those cases in which one focal paper is authored only by male scientists and the other focal paper is authored only by female scientists. Positive differences result when the male-authored paper of a pair has a higher share of male-authored citing papers than the female-authored paper of this pair. The histograms differ in the minimum cosine similarity between the tf-idf of two paired papers, and – as a consequence – in the number of pairs of focal papers included: all 455,434,277 pairs in (A), 29,648,614 pairs with a cosine similarity of at least 0.035 in (B), 8,631,470 pairs with a cosine similarity of at least 0.064 in (C), 2,252,018 pairs with a cosine similarity of at least 0.112 in (D), 546,588 pairs with a cosine similarity of at least 0.185 in (E), and 116,950 pairs with a cosine similarity of at least 0.287 in (F). The vertical lines are placed at 0 (black) and at the observed average difference (red, dashed). The black curve shows the shape of a normal distribution.

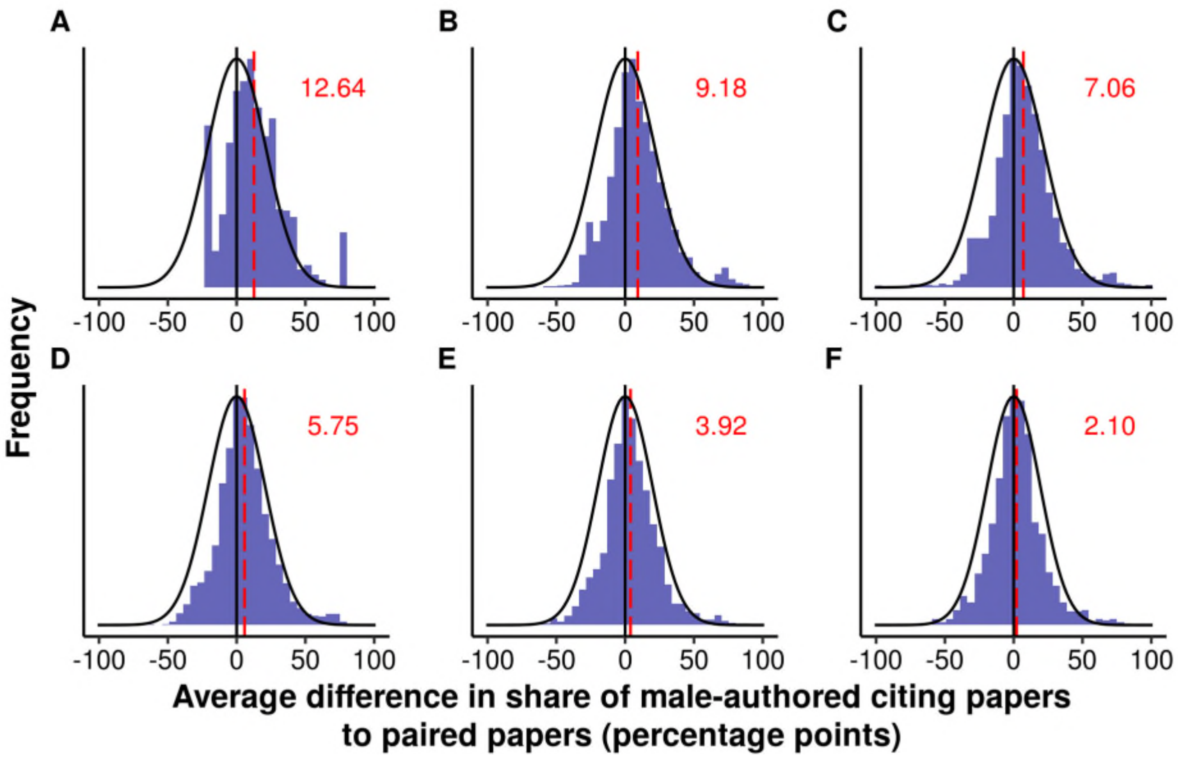

Fig. S14. Histograms for the male-authored focal papers' average difference in the share of male-authored citing papers to their paired female-authored focal papers (Faculty Opinions data). In each histogram, the male-authored focal papers are restricted to those that could be paired with at least one female-authored focal paper. Positive average differences result when the share of male-authored citations is higher for the male-authored focal paper than the average share for its paired female-authored papers. The histograms differ in the minimum number of shared keywords that the pairs of focal papers have, and – as a consequence – in the number of male-authored focal papers included: all 9,280 papers in (A), 9,150 papers that could be paired based on at least one shared Faculty Opinions keyword in (B), 7,533 papers that could be paired based on at least two shared Faculty Opinions keywords in (C), 5,062 papers that could be paired based on at least three shared Faculty Opinions keywords in (D), 2,644 papers that could be paired based on at least four shared Faculty Opinions keywords in (E), and 1,110 papers that could be paired based on at least five shared Faculty Opinions keywords in (F). The vertical lines are placed at 0 (black) and at the observed average difference (red, dashed). The black curve shows the shape of a normal distribution.

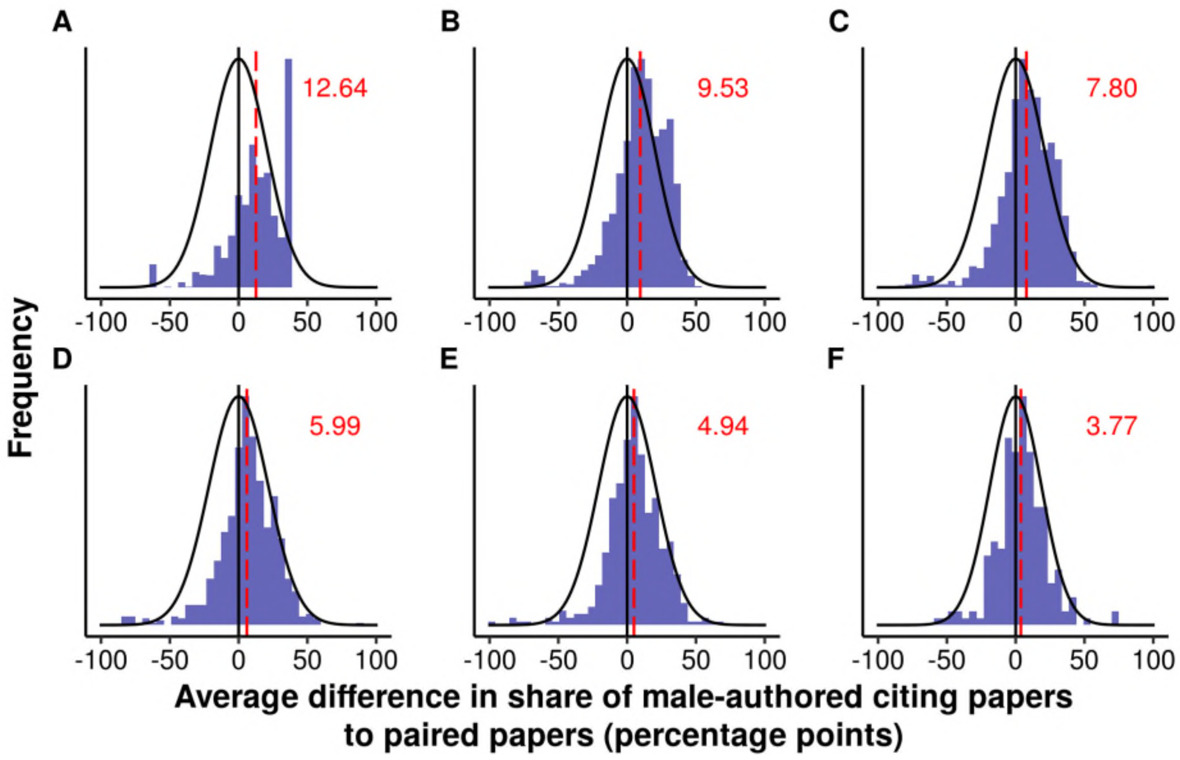

Fig. S15. Histograms for the female-authored focal papers' average difference in the share of male-authored citing papers to their paired male-authored focal papers (Faculty Opinions data). In each histogram, the female-authored focal papers are restricted to those that could be paired with at least one male-authored focal paper. Positive average differences result when the share of male-authored citations is smaller for the female-authored focal paper than the average share for its paired male-authored papers. The histograms differ in the minimum number of shared keywords that the pairs of focal papers have, and – as a consequence – in the number of female-authored focal papers included: all 1,261 papers in (A), 1,257 papers that could be paired based on at least one shared Faculty Opinions keyword in (B), 1,078 papers that could be paired based on at least two shared Faculty Opinions keywords in (C), 713 papers that could be paired based on at least three shared Faculty Opinions keywords in (D), 427 papers that could be paired based on at least four shared Faculty Opinions keywords in (E), and 210 papers that could be paired based on at least five shared Faculty Opinions keywords in (F). The vertical lines are placed at 0 (black) and at the observed average difference (red, dashed). The black curve shows the shape of a normal distribution.

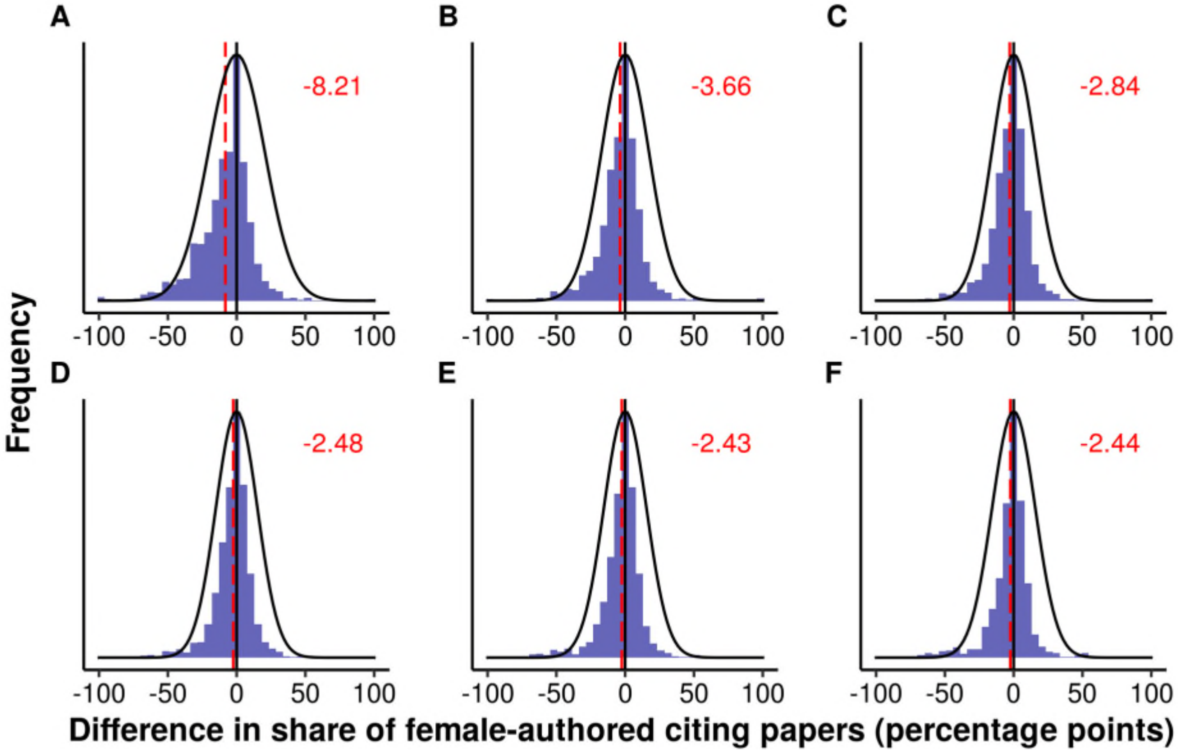

Fig. S16. Histograms for the differences in the share of female-authored citing papers for pairs of focal papers (Faculty Opinions data). In each histogram, the pairs of focal papers are restricted to those cases in which one focal paper is authored only by male scientists and the other focal paper is authored only by female scientists. Positive differences result when the female-authored paper of a pair has a higher share of female-authored citations than the male-authored paper of this pair. The histograms differ in the minimum number of shared keywords that the pairs of focal papers have, and – as a consequence – in the number of pairs of focal papers included: all 11,702,080 pairs in (A), 765,642 pairs with at least one shared Faculty Opinions keyword in (B), 223,837 pairs with at least two shared Faculty Opinions keywords in (C), 58,465 pairs with at least three shared Faculty Opinions keywords in (D), 14,167 pairs with at least four shared Faculty Opinions keywords in (E), and 3,010 pairs with at least five shared Faculty Opinions keywords in (F). The vertical lines are placed at 0 (black) and at the observed average difference (red, dashed). The black curve shows the shape of a normal distribution.

Table S1. Studies empirically analyzing gender homophily in citations.

Study	No. of citation links included	Selection of citing papers (number of papers / publication years / subfields)	Operationalization of gender	Controlling for subfields	Controlling for self-citations	Gender homophily rate
Ferber [16]	2,394	118 / 1982-1983 / economics (subfield manpower, labor and population)	Female-only author teams vs. male-only author teams	Only citing papers of one field included	Self-citations excluded	0.116
Ferber [17]	11,669	676 / 1982-1983 / economics, developmental psychology, mathematics, sociology	Female-only author teams vs. male-only author teams	Pairwise matching of papers based on fields	Self-citations excluded	0.081
Lutz [9]	10,593	446 / 1982-1986 / anthropology	First author	Only citing papers of one field included	Self-citations excluded	0.078
Davenport and Snyder [18]	4,951	100 / 1985-1994 / sociology	First author	Only citing papers of one field included	Not controlled	0.212
Mcelhinny, Hols, Holtzkener, Unger and Hicks [19]	16,766	Not available / 1965-2000 / sociolinguistic and linguistic anthropology	Author-author links considered	Only citing papers of one field included	Not controlled	0.133
Håkanson [10]	23,483	1,739 / 1980-2000 / library and information science	Female-only author teams vs. male-only author teams	Only citing papers of one field included	Self-citations excluded	0.124

Ferber and Brün [20]	3,256	238 / 2008 / economics (labor economics and general economics)	Female-only author teams vs. male-only author teams	Separate analysis for fields	Self-citations excluded	0.068
Knobloch-Westerwick and Glynn [21]	2,958	1,020 / 1991-2005 / communication science	First author	Only citing papers of one field included	Not controlled	0.16
Mitchell, Lange and Brus [22]	3,013	57 / 2005 / political science (international relations)	Female-only author teams vs. male-only author teams	Only citing papers of one field included	Not controlled	0.214
Potthoff and Zimmermann [23]	25,853	917 / 1970-2009 / communication science	Female-only author teams vs. male-only author teams (based on first two authors)	Only citing papers of one field included	Self-citations excluded	0.085
Dion, Sumner and Mitchell [24]	30,066	1,938 / 2007-2016 / political science and social science methodology	Female-only author teams vs. male-only author teams (based on first five authors)	Separate analysis for fields	Not controlled	0.102
Ghiasi, Mongeon, Sugimoto and Larivière [11]	20,395,382	1,557,967 / 2008-2016 / no restriction of fields	First & corresponding authors (same gender)	Pairwise matching of papers based on topics (abstract and title)	Self-citations excluded	0.099

Table S2. Regression results for pairs of focal papers (Faculty Opinions data).

	Dependent variable: difference in the share of male-authored citing papers
Number of shared Faculty Opinions keywords (reference category: 0)	
1	-4.566***
	(0.041)
2	-6.074***
	(0.073)
3	-7.737***
	(0.140)
4	-8.678***
	(0.279)
≥5	-10.942***
	(0.537)
Difference in quality ratings	2.098***
	(0.010)
Difference in age	0.375***
	(0.002)
Difference in team size	-0.665***
	(0.004)
Intercept	13.135***
	(0.009)
N	11,139,628
R^2	0.012

Note. Standard errors in parentheses.
* $p < 0.05$, ** $p < 0.01$, *** $p < 0.001$ (two-tailed test).

S3. References

1. Studer C. GitHub repository cstuder/genderReader. 2012(March 20, 2019):Retrieved from https://github.com/cstuder/genderReader.
2. Breusch TS, Pagan AR. A simple test for heteroscedasticity and random coefficient variation. Econometrica. 1979;47(5):1287-94.
3. Fox J, Monette G. Generalized collinearity diagnostics. Journal of the American Statistical Association. 1992;87(417):178-83.
4. James G, Witten D, Hastie T, Tibshirani R. An Introduction to Statistical Learning. With Applications in R: Springer; 2013.
5. Cook RD, Weisberg S. Residuals and Influence in Regression: Taylor & Francis; 1982.
6. Sammut C, Webb GI. TF–IDF. Encyclopedia of Machine Learning. Boston, MA: Springer US; 2010. p. 986-7.
7. Glänzel W, Thijs B, Schlemmer B. A bibliometric approach to the role of author self-citations in scientific communication. Scientometrics. 2004;59(1):63-77.
8. Backes T. The impact of name-matching and blocking on author disambiguation. Proceedings of the 27th ACM International Conference on Information and Knowledge Management. CIKM '18. Torino, Italy: ACM; 2018. p. 803-12.
9. Lutz C. The erasure of women's writing in sociocultural anthropology. American Ethnologist. 1990;17(4):611-27.
10. Håkanson M. The impact of gender on citations: An analysis of College & Research Libraries, Journal of Academic Librarianship and Library Quarterly. College & Research Libraries. 2005;66(4):312-23.
11. Ghiasi G, Mongeon P, Sugimoto CR, Larivière V. Gender homophily in citations. Proceedings of the International Conference on Science and Technology Indicators (STI) 2018. Leiden2018.
12. Thijs B. Science mapping and the identification of topics: Theoretical and methodological considerations. In: Glänzel W, Moed HF, Schmoch U, Thelwall M, editors. Springer Handbook of Science and Technology Indicators. Cham: Springer International Publishing; 2019. p. 213-33.
13. Garfield E. ISI's breakthrough retrieval method. Part 1. Expanding your searching power on Current Contents on Diskette. Essays of an Information Scientist: Journalology, KeyWords Plus, and other Essays. 131990. p. 295-9.
14. Garfield E, Sher IH. KeyWords Plus - algorithmic derivative indexing. J Am Soc Inform Sci. 1993;44(5):298-9.
15. Waltman L, Costas R. F1000 recommendations as a potential new data source for research evaluation: A comparison with citations. Journal of the Association for Information Science and Technology. 2014;65(3):433-45.
16. Ferber MA. Citations: Are they an objective measure of scholarly merit? Journal of Women in Culture and Society. 1986;11(2):381-9.

17. Ferber MA. Citations and networking. Gender & Society. 1988;2(1):82-9.
18. Davenport E, Snyder H. Who cites women? Whom do women cite?: An exploration of gender and scholarly citation in sociology. Journal of Documentation. 1995;51(4):404-10.
19. Mcelhinny B, Hols M, Holtzkener J, Unger S, Hicks C. Gender, publication and citation in sociolinguistics and linguistic anthropology: The construction of a scholarly canon. Language in Society. 2003;32(2):299-328.
20. Ferber MA, Brün M. The gender gap in citations: Does it persist? Feminist Economics. 2011;17(1):151-8.
21. Knobloch-Westerwick S, Glynn CJ. The Matilda effect - role congruity effects on scholarly communication: A citation analysis of Communication Research and Journal of Communication articles. Communication Research. 2013;40(1):3-26.
22. Mitchell SM, Lange S, Brus H. Gendered citation patterns in international relations journals. International Studies Perspectives. 2013;14(4):485-92.
23. Potthoff M, Zimmermann F. Is there a gender-based fragmentation of communication science? An investigation of the reasons for the apparent gender homophily in citations. Scientometrics. 2017;112(2):1047-63.
24. Dion ML, Sumner JL, Mitchell SM. Gendered citation patterns across political science and social science methodology fields. Political Analysis. 2018;26(03):312-27.
